# A comprehensive workflow for optimizing RNA-seq data analysis

**DOI:** 10.1186/s12864-024-10414-y

**Published:** 2024-06-24

**Authors:** Gao Jiang, Juan-Yu Zheng, Shu-Ning Ren, Weilun Yin, Xinli Xia, Yun Li, Hou-Ling Wang

**Affiliations:** 1 School of Information Science and Technology, School of Artificial Intelligence, Beijing Forestry University, Beijing, 100083 People’s Republic of China; 2 State Key Laboratory of Tree Genetics and Breeding, National Engineering Research Center of Tree Breeding and Ecological Restoration, College of Biological Sciences and Technology, Beijing Forestry University, Beijing, 100083 People’s Republic of China

**Keywords:** RNA-seq data, Differential gene analysis, Software comparison

## Abstract

**Background:**

Current RNA-seq analysis software for RNA-seq data tends to use similar parameters across different species without considering species-specific differences. However, the suitability and accuracy of these tools may vary when analyzing data from different species, such as humans, animals, plants, fungi, and bacteria. For most laboratory researchers lacking a background in information science, determining how to construct an analysis workflow that meets their specific needs from the array of complex analytical tools available poses a significant challenge.

**Results:**

By utilizing RNA-seq data from plants, animals, and fungi, it was observed that different analytical tools demonstrate some variations in performance when applied to different species. A comprehensive experiment was conducted specifically for analyzing plant pathogenic fungal data, focusing on differential gene analysis as the ultimate goal. In this study, 288 pipelines using different tools were applied to analyze five fungal RNA-seq datasets, and the performance of their results was evaluated based on simulation. This led to the establishment of a relatively universal and superior fungal RNA-seq analysis pipeline that can serve as a reference, and certain standards for selecting analysis tools were derived for reference. Additionally, we compared various tools for alternative splicing analysis. The results based on simulated data indicated that rMATS remained the optimal choice, although consideration could be given to supplementing with tools such as SpliceWiz.

**Conclusion:**

The experimental results demonstrate that, in comparison to the default software parameter configurations, the analysis combination results after tuning can provide more accurate biological insights. It is beneficial to carefully select suitable analysis software based on the data, rather than indiscriminately choosing tools, in order to achieve high-quality analysis results more efficiently.

**Supplementary Information:**

The online version contains supplementary material available at10.1186/s12864-024-10414-y.

## Introduction

RNA sequencing (RNA-Seq) is a technique used to determine the presence and abundance of RNA transcripts in specific biological samples at a particular time. It provides unprecedented detail about the RNA landscape [1], and comprehensive information about gene expression. This information also aids in understanding the regulatory networks, tissue specificity, and developmental patterns of genes involved in various biological processes. It enables the modeling and inference of signaling pathways to facilitate the biological applications [2]. Due to its wide applications in identifying new genes or transcripts, mutations, gene editing, and analyzing differential gene expression, RNA-Seq has gradually replaced microarrays as the primary method for transcriptome analysis [3–7].

Differential expression (DE) analysis is a primary objective of transcriptome analysis and involves several steps: trimming sequencing reads, alignment, quantification, and DE analysis [8, 9]. The trimming step aims to remove adapter sequences and low-quality nucleotides to improve read mapping rates. During the alignment step, reads are considered aligned if they correspond to specific regions on the reference genome or transcriptome. Sometimes, reads cannot be uniquely mapped due to repetitive sequences shared by paralogous genes within domains [10]. Alignment tools for RNA-Seq typically include customizable thresholds to accommodate mismatches during alignment caused by sequencing errors or biological variations such as mutations [11]. Handling repetitively aligned or incompletely aligned reads is crucial for enhancing the accuracy and reliability of analysis results.

The quantification step determines the number of reads mapped to each genomic region using annotation files that correspond to the reference genome. Depending on the biological sample and research objectives, suitable features from three levels—genes, transcripts, exons—can be selected for gaining count matrix. The DE analysis aims to provide more biological insights into the genetic mechanisms underlying phenotypic differences by identifying genes that exhibit differential expression patterns under different conditions, in conjunction with downstream analyses. Due to the differences in data distribution theories corresponding to analysis methods, the common theoretical distributions of RNA-seq reads are the Poisson distribution and the negative binomial distribution. Modifying normalization parameters, hypothesis testing parameters, and fitting parameters in different DE methods are key considerations for users [12–15].

With the widespread application of RNA-seq, numerous analysis tools have been developed [16]. However, they involve various programming languages and operating platforms, making it challenging for researchers without relevant expertise [17]. Users also face the challenge of constructing a complete workflow in a specific analysis order and selecting from a complex methodology [18, 19]. The design of the analysis pipeline needs to consider the sequencing technology used in the project, sample types, focus of analysis, and availability of computational resources [20]. Different analysis methods have varying emphases and computational requirements, resulting in significant differences in accuracy, speed, and cost across various workflows [21]. Therefore, it is crucial to investigate how different steps affect the analysis results. Despite extensive research conducted by scholars so far on analyzing RNA-seq data for optimal methods, a consensus has not yet been reached [16–18, 20–22].

Several studies have been performed to evaluate and compare the performance of different RNA-seq analysis tools [10, 20–24]. However, comprehensive and systematic analyses from different perspectives are still lacking because most workflow analyses only focus on several steps or primarily use human data [25–31]. The best-performing workflow based on existing metrics may not ensure optimal performance across all datasets, this relies on extensive validation experiments using diverse datasets. However, although RNA-seq has generated a vast amount of experimental data due to its widespread use, a recent found that a mere 25% of articles outline all crucial computational procedures, with an even smaller fraction providing detailed parameter values necessary for achieving full reproducibility [19]. The lack of its complete announcement of analysis parameters of the whole workflow making their results unsuitable for validating performance across different workflows [32]. Currently, there is still a lack of appropriate metrics to evaluate the performance of various methods [33]. It’s is needed to compare these methods to achieve optimal accuracy within cost and performance constraints for RNA-seq processing.

Fungi play an important role in natural ecosystems by participating in ecological processes such as organic matter decomposition and cycling. However, fungi can also negatively affect the ecological and economic value of plants. Fungal diseases account for a significant proportion of plant diseases, estimated at 70%-80%, adversely affecting agricultural and forestry crop yields and quality [34]. With the development of high-throughput sequencing technologies, RNA-seq has become a common method for researchers studying fungal diseases [35]. Transcriptome analysis identifies disease resistance genes [36] and related pathways [37], providing a foundation for resistant breeding [38–40]. Studying the mechanisms of interaction between fungi and plants enables the development of more effective biological control strategies [41, 42], while also reducing environmental pollution. Existing RNA-seq analysis software lacks species specificity because the statistical and analytical parameters used are typically consistent across different organisms, including humans, animals, plants, fungi, and bacteria. This may compromise the applicability and accuracy of analyses.

In consideration of this deficiencies for RNA-seq data analysis, our study addresses these limitations by investigating the impacts of different parameters at each step of the analysis. According to the classification in the former reports [43, 44], it is understood that plant-pathogenic fungi mainly distribute across the phyla *Ascomycota, Basidiomycota, Blastocladiomycota, Chytridiomycota, and Mucoromycota* in the fungal evolutionary tree. The three datasets we chosed (*Magnaporthe oryzae, Colletotrichum gloeosporioides and Verticillium dahliae*), all belong to the *Pezizomycotina* subphylum under the *Ascomycota* phylum (Supplementary Figs. 1 and 2). Another significant branch under the *Ascomycota* phylum in the fungal evolutionary tree, the *Saccharomycotina* subphylum, lacks plant-pathogenic fungi and thus is not considered. In order to enhance the representativeness of plant-pathogenic fungi data in this study, we also used transcriptome data of *Ustilago maydis* and *Rhizopus stolonifer*. They belong to the *Ustilaginomycotina* and *Agaricomycotina* + *Wallemiomycotina* branches, respectively, which constitute the second-largest group in the fungal evolutionary tree, *Basidiomycota* phylum (Supplementary Figs. 1 and 2). This ensures that this study encompasses the major species of plant-pathogenic fungi.

**Fig. 1:**
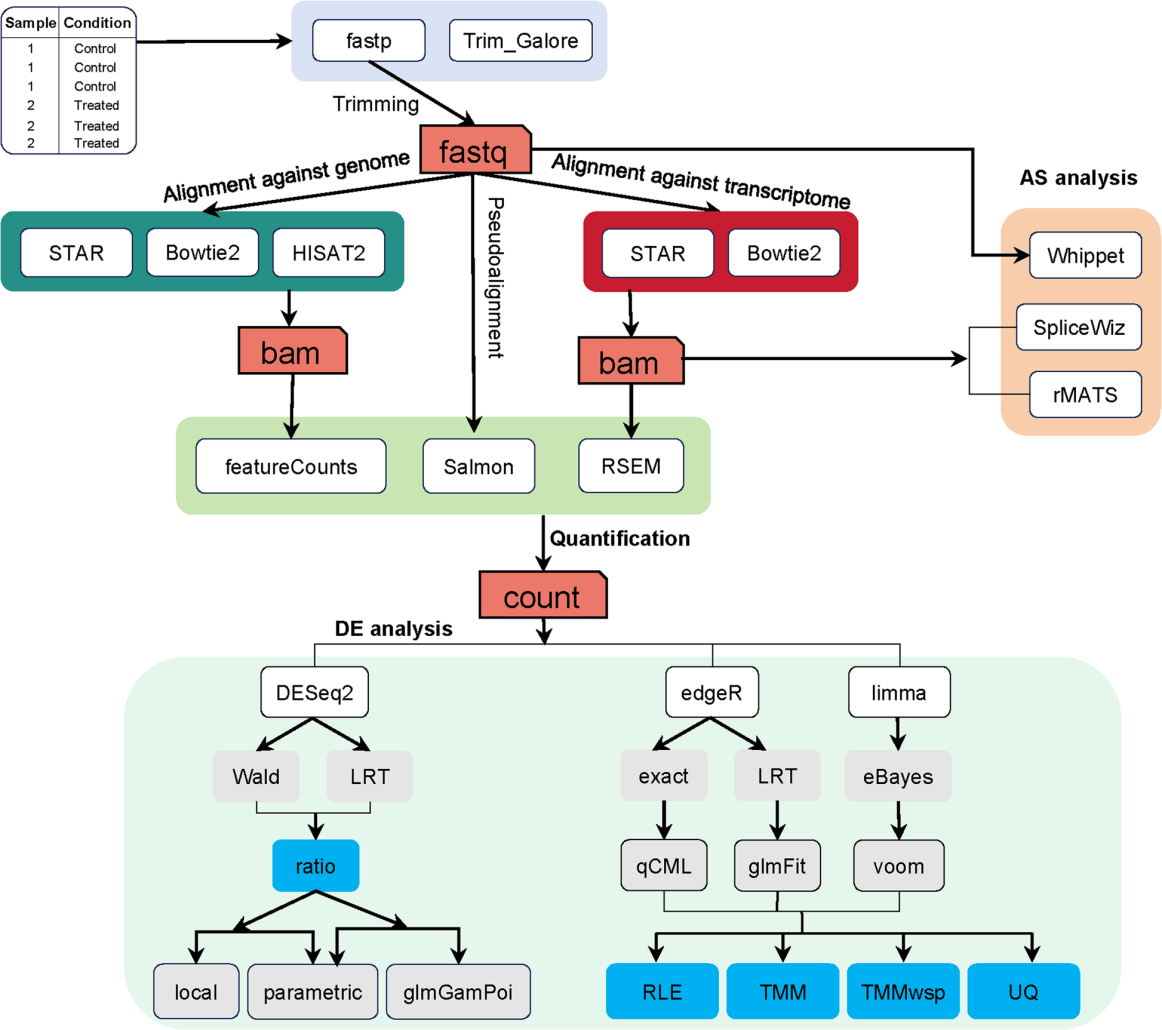
RNA-seq analysis workflow. The different colored boxes represent distinct stages of analysis, with white boxes representing the software used in each stage. The arrows depict the combination relationships between different software

In this study, software selection was guided by two main principles: choosing tools widely used in transcriptome analysis and considering researchers' preferences for operational simplicity or feature richness during the analysis process. We compiled the citation counts of publications associated with tools across various stages, as documented in Google Scholar (due to May 4, 2024), to assess their prevalence and adoption in the field (Supplementary Table 1). The analytical tools utilized in this study and the workflow derived from their combination are depicted in Fig. 1. The detailed description of the criteria and reason used for tool selection can be found in the “Results” section. Moreover, we conducted additional validation using datasets from animal species (mice, *Mus musculus*) and plant species (poplar, *Populus tomentosa*) to validate our findings. Our study optimizes the analysis process based on the results obtained for the analysis of differential gene expression in RNA-seq data. Through this investigation, we present a relatively user-friendly workflow for RNA-seq analysis, which can help individuals gain valuable insights into RNA-seq data analysis.

## Results

### Filtering and trimming

Commonly utilized tools for filtering and trimming stages include fastp [45], Trimmomatic [46], Cutadapt [47], and Trim_Galore [48]. Considering researchers’ tendencies to favor either straightforward operation or feature-rich integrated tools during analysis,we utilized two commonly used tools for filtering and trimming, namely fastp [45] and Trim_Galore [48]. The former is advantageous due to its rapid analysis and simplicity of operation, while the latter has become a preferred analysis tool for many researchers because it can generate quality control reports concurrently with filter and trimming process. Trim_Galore integrates Cutadapt [47] and FastQC [49] for comprehensive quality control(QC) analysis in a single step, so we did not duplicate the comparison with Cutadapt. Despite Trimmomatic being the most cited QC software, its parameter setup is complex and it does not offer a speed advantage. Considering the foundational nature of QC software usage in this study, we therefore did not select Trimmomatic [46]as a research tool.

To investigate the impact of trimming parameters on data quality, we compared the effects of these parameters on the proportions of Q20 and Q30 bases, as well as their influence on the alignment rate in subsequent alignment process. When setting the parameter for the number of bases to be trimmed, instead of directly specifying numerical values as done previously, we chose two base positions, FOC and TES, for trimming based on the quality control report of the original data (refer to the Method section for details). The trimming parameters of each dataset are shown in Supplementary Table 2. Although Trim_Galore enhanced the quality of bases, it led to an unbalanced base distribution in the tail (Supplementary Fig. 3). Despite making several attempts with different datasets and adjusting adapter parameters based on recommendations from the community, the problem persisted when using Trim_Galore.

**Fig. 2:**
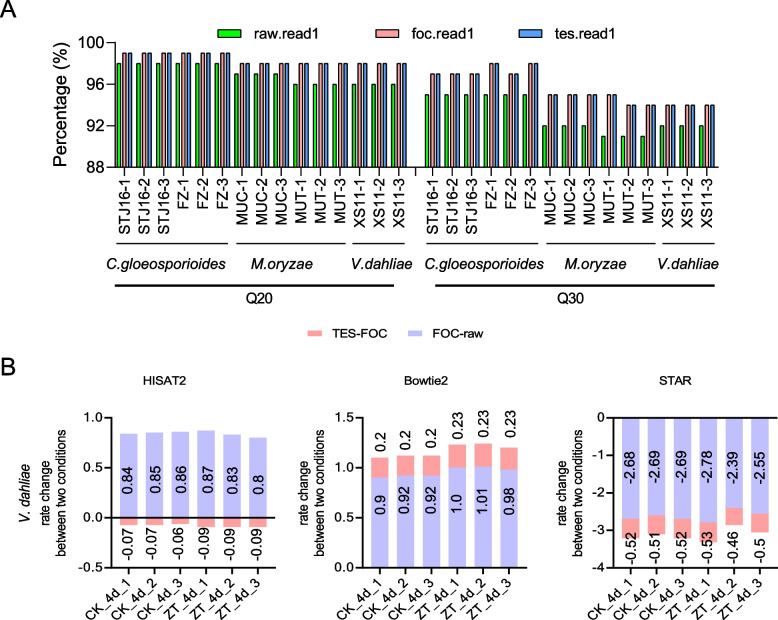
Comparison of analysis results of different software. **A** The histogram of Q20 and Q30 base content percentage of filter results obtained by using fastp software under three clipping parameters (raw, FOC, TES) using three fungal data, respectively. **B** The bar chart illustrates changes in alignment rates (*V. dahliae* data set). Bar length corresponds to the magnitude of change, with positive values indicating an increase and negative values indicating a decrease in alignment rates. The pink bars (TES-FOC) depict alignment rate changes between TES processing and the FOC processing states, while the blue bars (FOC-raw) depict changes between the FOC processing and raw processing states

In terms of filtering and trimming effects, fastp significantly enhanced the quality of the processed data (Fig. 2A, Supplementary Table 3). Compared with the FOC treatment, the proportion of Q20 and Q30 bases after TES treatment was almost zero, while the base quality improvement after FOC treatment ranged from 1 to 6%. In this study, the parameter values of FOC and TES differ by 1–5, but there is almost no difference in processing results, indicating that excessive trimming did not substantially enhance the quality of sequencing data.

**Fig. 3:**
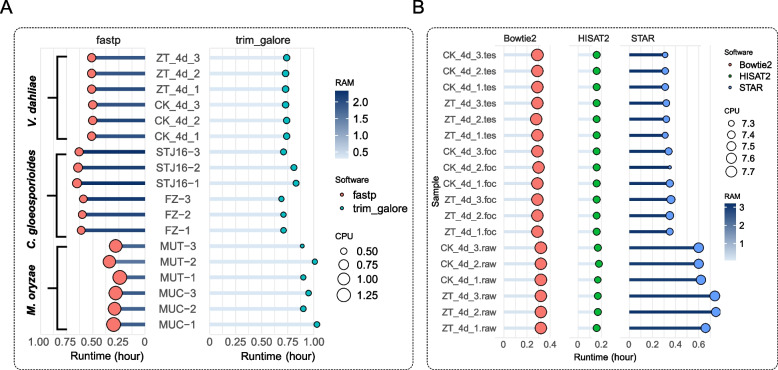
Runtime, CPU and RAM occupancy of different tools. **A** Comparison between Trim_Galore and fastp. **B** Comparison between different aligners under the same running threads

Thus, when dealing with these data, choosing FOC as the trimming parameter during the filtering and trimming stage is a more advantageous optimal choice. In terms of processing speed (Fig. 3A, Supplementary Fig. 4), fastp demonstrated superior performance compared to Trim_Galore. The computational efficiency of fastp is approximately 1.5 to 4 times faster than Trim_Galore, despite consuming 2–4 times more RAM (around 2G). This resource utilization remains within acceptable bounds for individual analytical users in academic research settings. In summary, fastp demonstrates greater efficiency and stability compared to Trim_Galore, establishing it as the recommended choice for this processing step.

### Alignment

Bowtie [50], Bowtie2 [51], TopHat [52], TopHat2 [53], HISAT [54], HISAT2 [55], STAR [56] are commonly used tools for alignment. Although Bowtie and Bowtie2 have similar names, they are two different types of alignment tools. Bowtie2 is more suitable for aligning longer reads, aligning better with current sequencing technology trends. Tophat and Tophat2 use Bowtie and Bowtie2 as their alignment foundation, respectively. The HISAT series and Tophat series were developed by the same laboratory. The authors recommend using the latest HISAT2 to replace HISAT and Tophat2, and the Tophat series is no longer maintained. STAR is renowned for its efficient analysis capabilities and is widely used in transcriptome analysis. Based on these considerations, we selected HISAT2, STAR, and Bowtie2 as the alignment phase analysis tools in our study.

Given the variations in the efficacy of quality enhancement during the trimming and filtering stage of sequencing data, a more in-depth investigation of the alignment rates associated with these processes is required. In the fungal dataset, when focusing solely on alignment rates, we found that three datasets (*V. dahliae, M. oryzae and U. maydis*) exhibited the highest performance under the FOC trimming treatment corresponding to HISAT2 among the three states (Fig. 2B, Table 1, Supplementary Fig. 5A, 6A), while Bowtie2 and STAR method did not consistent with this pattern under the FOC treatment state (Fig. 2B, Supplementary Fig. 5B-C, Supplementary Fig. 6). When using Bowtie2, it was consistently observed that the TES treatment results in the highest alignment rates across datasets, indicating that a greater degree of base trimming corresponds to an elevated alignment rate. Conversely, in the case of STAR, reduced base trimming was associated with higher alignment rates, except for the *C. gloeosporioides* data set. However, from the perspective of the improvement in the read mapping rate, the conclusion of the mapping step was consistent with that of the previous trimming and filtering step, indicating that appropriate trimming is preferable.

**Table 1:** Summary of the alignment rate

	HISAT2			Bowtie2			STAR		
M. oryzae	raw	FOC	TES	raw	FOC	TES	raw	FOC	TES
MUC-1	87.54	89.44	88.65	83.98	86.71	87.09	94.04	92.45	91.41
MUC-2	88.95	91.04	90.38	84.7	87.91	87.55	94.5	93.59	92.74
MUC-3	88.41	90.32	89.72	84.59	87.14	87.5	94.36	93.13	92.34
MUT-1	87.8	92.24	91.94	84.61	86.97	87.3	93.88	94.85	94.4
MUT-2	85.98	89.55	88.92	83.57	86.34	86.71	93.09	92.72	91.86
MUT-3	86.31	89.91	89.33	83.75	86.51	86.86	93.18	92.95	92.15
C. *gloeosporioides*	raw	FOC	TES	raw	FOC	TES	raw	FOC	TES
FZ-1	86.7	89.49	89.76	83.34	85.1	85.45	92.39	92.78	92.27
FZ-2	85.92	89.21	89.49	83.17	85.11	85.48	91.97	92.85	92.5
FZ-3	85.21	88.32	88.59	82.79	84.76	85.1	90.65	91.49	91.08
STJ16-1	85.66	87.87	88.16	79.37	80.92	81.35	90.7	91.33	91.2
STJ16-2	85.99	88.07	88.35	79.91	81.38	81.81	91.17	91.72	91.55
STJ16-3	85.47	87.76	88.06	79.38	81	81.44	90.65	91.27	91.11
V. dahliae	raw	FOC	TES	raw	FOC	TES	raw	FOC	TES
CK_4d_1	93.3	94.14	94.07	89.07	89.97	90.17	95.48	92.8	92.28
CK_4d_2	93.53	94.38	94.31	89.19	90.11	90.31	95.53	92.94	92.43
CK_4d_3	93.53	94.39	94.33	89.18	90.1	90.3	95.48	92.79	92.27
ZT_4d_1	93.36	94.23	94.14	88.13	89.13	89.36	95.59	92.81	92.28
ZT_4d_2	93.3	94.13	94.04	87.93	88.94	89.17	95.45	93.06	92.6
ZT_4d_3	93.41	94.21	94.12	87.98	88.96	89.18	95.5	92.95	92.45
XS11_4day_1	80.85	89.87	89.88	78.38	85.92	86.03	85.42	85.83	85.57
XS11_4day_2	80.13	88.6	88.6	79.7	86.89	86.98	85.91	85.46	85.22
XS11_4day_3	76.28	83.84	83.89	79.87	86.48	86.58	86.65	87.34	87.08

To further validate our findings, experiments were carried out utilizing data from mice (*M. musculus*) and poplar (*P. tomentosa*) data. The results revealed that, in contrast to the fungal datasets, the transcriptome data of poplar exhibited a higher alignment rate with HISAT2 and Bowtie2 as the number of trimmed bases rose (Supplementary Fig. 7A-B). Additionally, when using STAR, the sequencing files under FOC treatment demonstrated the highest alignment rate (Supplementary Fig. 7C), which differed from the fungal data. The analysis of the mice (*M. musculus*) data yielded outcomes akin to poplar when using Bowtie2, whereas the results obtained with HISAT2 and STAR were found to be analogous to certain fungal datasets (Supplementary Fig. 7D-F). This indicated that different alignment software indeed exhibits certain variations when handling data from different species. A detailed comparison of the read pairs and read alignment is presented in Supplementary Fig. 8 and Supplementary Fig. 9. Remarkably, Bowtie2 consistently exhibited the highest proportion of read pairs that were uniquely mapped, encompassing both those that were uniquely mapped and unmapped. The other two software showed higher occupation of one pair unique mapped and corresponding pair unmapped. STAR exhibited the highest proportion of unique mapping reads without errors, with the proportion of other categories being almost negligible. HISAT2 showed a lower proportion of unique mapping without errors, whereas Bowtie2 exhibited the lowest result. This detailed comparison has provided us with a clearer understanding of the analytical distinction among different alignment software.

Instances have arisen where the processing time of the same software, with an identical thread count of 32, varied by a factor of six, surpassing even the time taken even when the thread count was set to 8 or 16(Table 2). According to Table 2, HISAT2 demonstrated the quickest processing time in analyzing the raw fastq of *V. dahliae* dataset, completing the task in just 0.5 h. Subsequently, STAR required 1.7 h for the analysis, while Bowtie2 exhibited the slowest performance, taking 2.5 h to complete the process. The analysis of CPU utilization rates from common batch running codes (with consistent parameters) revealed significant time difference despite setting identical thread counts for our programs. To enhance the assessment of the performance of each software in terms of running speed, we adjusted the experimental code to guarantee an equal number of threads for each software. In our investigation, HISAT2 consistently demonstrated superior performance compared to the other two software options, achieving an average runtime of 0.15 h. While Bowtie2 and STAR exhibited similar processing speeds (Fig. 3B, Supplementary Fig. 10), their runtimes were typically 2–3 times longer than that of HISAT2. However, it is noteworthy that STAR consumed the highest RAM usage among the three software, approximately three times more than the other two tools.

**Table 2:** Summary of the computer resources utilization for alignment tools

		HISAT2			Bowtie2			STAR		
		threads	CPU (%*100)	time(h)	threads	CPU (%*100)	time(h)	threads	CPU (%*100)	time(h)
*M. oryzae*	raw	32	0.89	6.82	8	7.91	0.80	8	2.60	2.14
	FOC	32	2.00	1.03	32	1.21	9.17	8	1.69	1.54
	TES	32	2.01	0.90	32	1.19	9.03	8	10.19	0.22
		HISAT2			Bowtie2			STAR		
*C. gloeosporioides*	raw	16	10.19	0.22	8	2.03	3.30	8	1.23	2.53
	FOC	16	10.95	0.18	16	2.08	2.57	8	1.65	1.42
	TES	16	8.35	0.23	8	0.40	7.43	8	1.54	1.18
		HISAT2			Bowtie2			STAR		
*V. dahliae*	raw	8	5.30	0.52	8	5.14	2.50	8	5.33	1.70
	FOC	8	5.30	0.49	8	5.70	1.48	8	5.09	0.95
	TES	16	1.23	5.68	16	1.21	6.01	8	4.01	0.78

*The mean values of CPU usage and time (in hours) were computed for each dataset

### Alternative splicing

The top four alternative splicing (AS) analysis tools, rMATS [57], MISO [58],VAST Tools [59] and DEXseq [60] (exon-based), were determined by the frequency of citations on Google Scholar. Whippet [61] and VAST Tools are research outcomes originating from the same laboratory but released at different times. Previous research has demonstrated the strong performance of Whippet in the field of AS analysis, hence it was chosen for experiments in this study. For choosing alternative splicing (AS) analysis tools, we excluded tools with limited analytical capabilities, MISO, and focused mainly on event-based tools, excluding DEXeq. Furthermore, contemporary tools for AS analysis were integrated, incorporating recent advancements like SpliceWiz [62], thereby enriching the comprehensiveness of this research.

In terms of computational efficiency, the execution time of an AS workflow using rMATS was approximately 1–2 times longer than that of a Whippet workflow. Conversely, the computational time required for running SpliceWiz was roughly double that of rMATS. When evaluating the total count of alternative splicing (AS) events, it is evident that rMATS and SpliceWiz possessed an undeniable competitive edge (Tables 3, 4 and 5). While thousands of AS events were identified, Whippet produced only a limited number of results. The results of AS analysis using rMATS combined with Bowtie2 are significantly different compared to those obtained using the other two alignment software in our fungal datasets. To verify whether the performance of the pipeline combining Bowtie2 and rMATS was poor only on fungal datasets, experiments were conducted on plant (*P. tomentosa*) and animal (*M. musculus*) datasets. The results showed that the AS analysis pipeline combining Bowtie2 did not entirely perform poorly on animal and plant datasets, as shown in Supplementary Table 4. Although SpliceWiz can import BAM files after the alignment process, the resulting BAM files from Bowtie2 are not compatible. In addition, while SpliceWiz can handle input FASTQ files, it is restricted to alignment processing using STAR. It is worth noting that the alignment mode of SpliceWiz was not used in this study. Instead, all analyses were performed based on the outputs of the three software tools used in the alignment process.

**Table 3:** Summary of the results of rMATS

M. oryzae	SE			RI			A3SS			A5SS			MXE		
	raw	FOC	TES	raw	FOC	TES	raw	FOC	TES	raw	FOC	TES	raw	FOC	TES
Bowtie2	6	6	6	37	37	37	10	10	10	17	17	17	0	0	0
HISAT2	1582	1507	1483	38	39	39	23	21	21	30	30	30	213	190	187
STAR	1805	1643	1593	40	39	39	25	23	23	30	30	30	273	223	210
C. gloeosporioides	SE			RI			A3SS			A5SS			MXE		
	raw	FOC	TES	raw	FOC	TES	raw	FOC	TES	raw	FOC	TES	raw	FOC	TES
Bowtie2	0	0	0	0	0	0	0	0	0	0	0	0	0	0	0
HISAT2	528	500	491	0	0	0	0	0	0	0	0	0	27	24	23
STAR	569	518	506	0	0	0	0	0	0	0	0	0	32	26	25
V. dahliae	SE			RI			A3SS			A5SS			MXE		
	raw	FOC	TES	raw	FOC	TES	raw	FOC	TES	raw	FOC	TES	raw	FOC	TES
Bowtie2	0	0	0	0	0	0	0	0	0	0	0	0	0	0	0
HISAT2	1441	1396	1387	0	0	0	0	0	0	0	0	0	227	220	216
STAR	1455	1404	1394	0	0	0	0	0	0	0	0	0	235	225	221

**Table 4:** Summary of the results of splicewiz

M. oryzae	SE			RI			A3SS			A5SS			MXE		
	raw	FOC	TES	raw	FOC	TES	raw	FOC	TES	raw	FOC	TES	raw	FOC	TES
HISAT2	1	1	1	717	616	591	2	2	2	4	3	3	0	0	0
STAR	1	1	1	744	643	615	2	2	2	3	3	2	0	0	0
C. gloeosporioides	SE			RI			A3SS			A5SS			MXE		
	raw	FOC	TES	raw	FOC	TES	raw	FOC	TES	raw	FOC	TES	raw	FOC	TES
HISAT2	0	0	0	1530	1382	1346	5	10	10	7	9	9	0	0	0
STAR	0	0	0	771	657	623	3	3	3	2	2	2	0	0	0
V. dahliae	SE			RI			A3SS			A5SS			MXE		
	raw	FOC	TES	raw	FOC	TES	raw	FOC	TES	raw	FOC	TES	raw	FOC	TES
HISAT2	2	2	3	1115	1030	1019	14	12	12	11	13	13	0	0	0
STAR	2	2	2	1141	1031	1017	14	10	9	16	13	14	0	0	0

**Table 5:** Summary of the results of whippet

M. oryzae					C. gloeosporioides					V. dahliae				
	SE	RI	A3SS	A5SS		SE	RI	A3SS	A5SS		SE	RI	A3SS	A5SS
raw	47	19	1	4	raw	244	0	0	0	raw	7	0	0	0
FOC	49	17	2	6	FOC	253	0	0	0	FOC	7	0	0	0
TES	46	17	2	5	TES	251	0	0	0	TES	7	0	0	0

For rMATS and SpliceWiz, a higher level of sequence retention led to an increased number of AS events being identified. Specifically, the original sequences without any trimming had the highest count of events. In contrast, Whippet demonstrated optimal performance when the data underwent appropriate trimming. A comparative analysis revealed no concurrence in the outcomes produced by rMATS and Whippet. Notably, Whippet is not align-based, relying on the fastq files of sequenced samples and reference genome annotation files. The unsatisfactory results obtained by Whippet in this study may be attributed to the lack of high-quality GTF annotation files in fungal data. Previous research has indicated that there is a limited yet existing overlap in the results produced by various software [63]. The anomaly observed in this study could be associated with the abnormal results of the Whippet analysis.

SpliceWiz and rMATS had similar change tendency. However, there was a notable discrepancy in the distribution of alternative splicing event categories between these two software. SpliceWiz excelled in detecting intron retention (IR) events, while rMATS demonstrated proficiency in identifying skipped exon (SE) and mutually exclusive exons (MXE) events. To know ground truth, we used AsimulatoR tool to simulate the whole genome of *V.dahliae*. Moreover, we compared the alternative splicing event conducted by STAR (Additional file 6, Supplementary Fig. 11). The overall results and results categorized by DAS (differential alternative splicing) event type for each tool were examined, as shown in Supplementary Fig. 12.

We found that both simulated data and real data had similar tendency. In simulation results, rMATS exhibited the highest precision in detecting MXE events, while rMATS showed the highest overall recall. Whippet also demonstrated relatively high precision, albeit analyzing fewer events, and only identified SE events in *V.dahliae* data. Similarly, SpliceWiz only detected IR events. Consistent with the real data scenario, the results obtained from validation with simulated data also revealed biases in event types detected by different tools. rMATS showed strong discriminatory ability for MXE and SE events, while Whippet and SpliceWiz performed well in detecting SE and IR events, respectively.

### Quantification

In quantitative analysis, software can be broadly categorized into two main groups: alignment-based transcript quantification tools, such as featureCounts [64], HTseq [65], and RSEM [66], and alignment-free transcript quantification tools, including Salmon [67] and Kallisto [68]. We did not choose HTSeq due to its longer analysis time and lack of clear advantages compared to other tools. The software featureCounts is widely used for quantitative analysis due to its rapid processing speed, while RSEM can simultaneously perform both alignment and quantification tasks. Previous studies have demonstrated that alignment-based quantification tools yield higher accuracy in results [29]. In order to gain a deeper understanding of alignment-based quantification tools, our study selected RSEM and featureCounts for comparative analysis within this category. Among non-alignment quantification tools, Kallisto and Salmon are the most widely used. We chose Salmon, which has higher citation rates and was released later, to compare with other quantification tools.

**Fig. 4:**
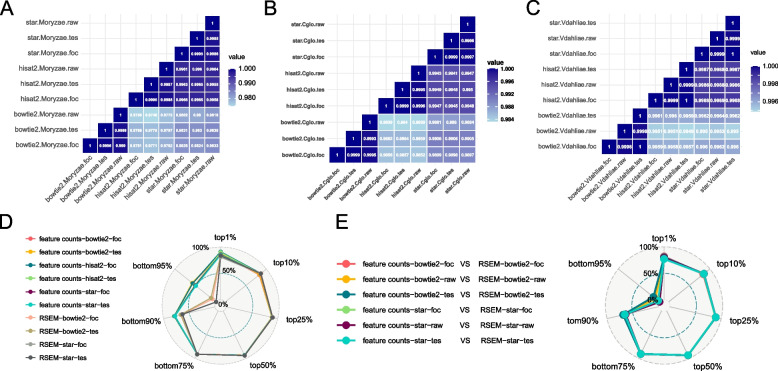
Calculations of different quantification results. Heatmap of different schemes using feature counts as quantification tool based on the Spearman rank correlation of their log expressions in **A** *M. oryzae*, **B** *C. gloeosporioides*, **C** *V. dahliae* datasets. **D** Correlation between the quantification results of different trimming and filtering treatment, both using raw data as benchmark data. **E** Correlation between the quantification results of different quantification tools

In this step, we changed all the quantification results into gene level, so that comparison can be carried out from the consistent level. For quantitative results, data generated through the utilization of the identical alignment software exhibited a strong correlation (Fig. 4A-C, Supplementary Fig. 13), consistently exceeding 0.98. It was found that there was little variance in the quantitative results obtained by different workflow. The simplicity of the fungal data structure in comparison to the RNA-seq data of other species like humans, animals, and plants may account for this phenomenon.

In Fig. 4 D and E, additional investigations demonstrated that the observed low correlations in both different upstream processing methods and results obtained from different quantitative software were primarily attributed to genes with low expression levels (genes falling within the 95th percentile). Interestingly, when examining various workflows, genes located within the 50th percentile always exhibited the most stable gene expression patterns (Supplementary Fig. 14).

### Simulation of read count

**Fig. 5:**
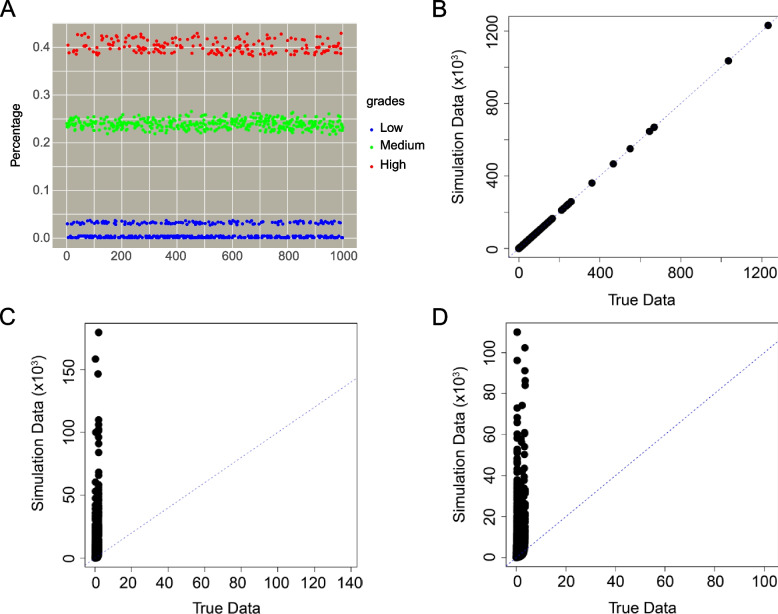
The analysis of count data simulation. **A** the Scatter plot of the multiple data simulations. **B** the Q-Q plots between the real data and the simulated data nearly corresponding to the true DE proportions. **C**,**D** the Q-Q plots between the real data and the simulated data with wrong DE proportions

Several data simulation tools were evaluated, and based on its favorable performance [69], the decision was made to utilize seqgendiff [70]. In the construction of the simulated datasets, 1,000, 2,000, and 5,000 repeated simulations were conducted, employing the same authentic dataset but adjusting the parameters of the resampling method. Interestingly, regardless of the variations in parameters, the percentage of differentially expressed genes in the simulated data consistently displayed a three-tiered distribution (Fig. 5A). Furthermore, only one exhibited a proportion of differentially expressed genes comparable to that of the real data set (Fig. 5B). Intriguingly, the association between the proportion in each level and the experimental order appeared stochastic.

**Fig. 6:**
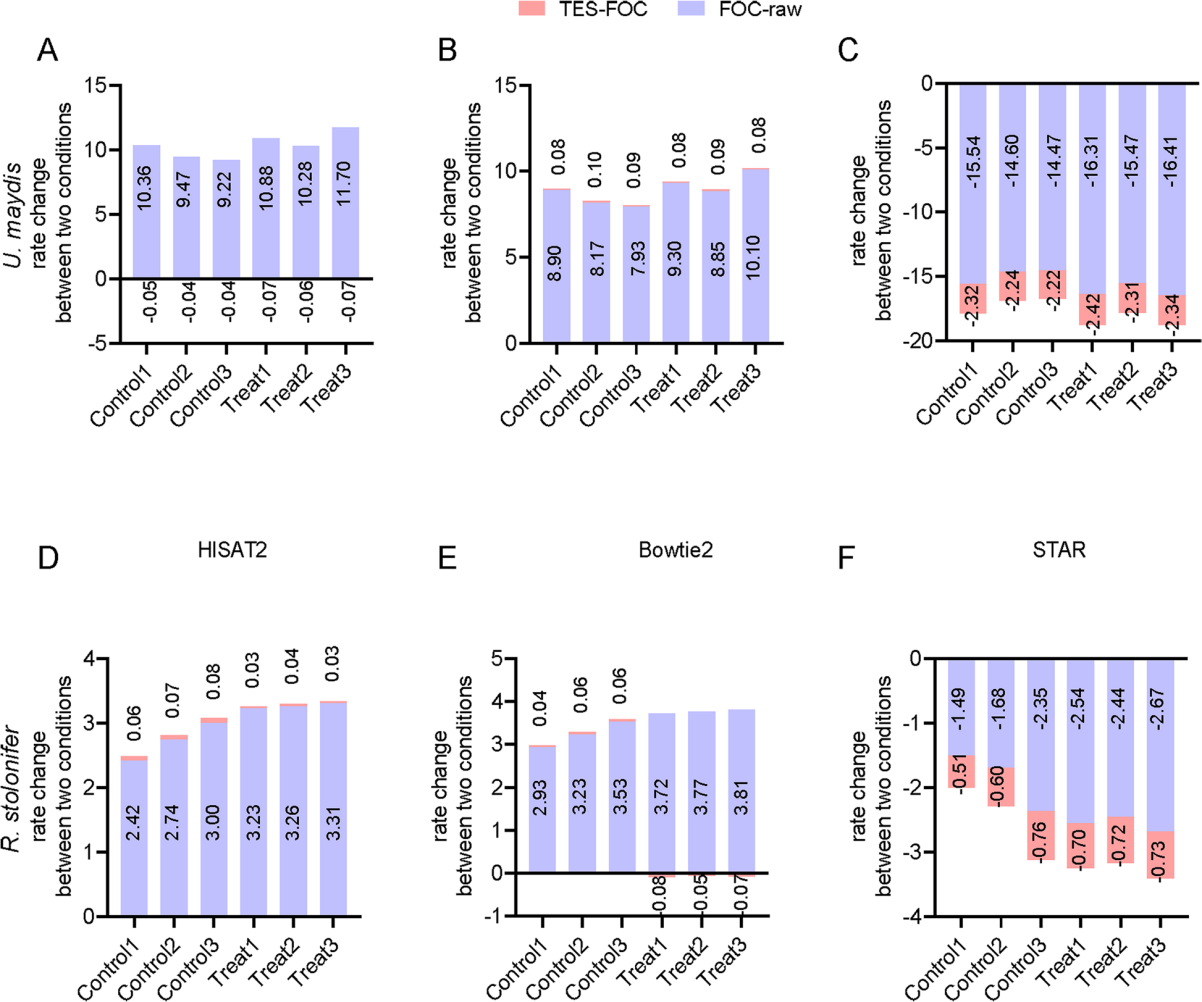
The analysis of count data simulation. **A** Workflow of data simulation. The violin-plot of different normalization methods in **B** *V. dahliae*, **C** *C. gloeosporioides*, and **D** *M. oryzae* datasets

Subsequently, we explored two datasets where the proportion of differentially expressed genes identified through data simulations significantly deviated from the real data. Notably, the distribution patterns in these datasets were markedly disparate from those observed in the real data (Fig. 5C-D). To ensure that the simulated data accurately reflects the authentic distribution, we refined the workflow of the data simulation process (Fig. 6A).

### Differential gene expression analysis

Among the commonly used software for analyzing differential gene expression, edgeR [71], DESeq2 [72], limma [73], Cuffdiff2 [74], baySeq [75] belong to parametric methods, while NOISeq [76] and SAMSeq [77] are categorized as non-parametric methods. This classification can also be further refined based on other attributes, such as whether it is count-based, the programming language utilized, and additional classification criteria. For differential gene analysis software, DESeq2, edgeR, and limma are widely cited and have clear advantages. DESeq2 and edgeR both employ a negative binomial distribution model for differential expression analysis, which takes into account the over-dispersion inherent in the data, thus enhancing the reliability and robustness of the results. On the other hand, limma utilizes linear models and empirical Bayes methods, improving the accuracy and stability of the analysis through model scaling. These three tools boast extensive user communities and abundant documentation, facilitating accessibility to tutorials for beginners and swift resolution of usage-related issues. Thus, we chose these three software packages for differential gene analysis. In addition to using the default settings, users are also given the option to modify the relevant parameters themselves. The software allows for the adjustment of parameters categorized into normalization method, fitting method, and hypothesis testing method. Sixteen differential expression analysis methods were obtained based on the variations in these three types of parameters (Fig. 1 and Supplementary Table 5).

We employed the Kruskal–Wallis rank sum test to evaluate the variations in data resulting from seven standardization methods. Our goal was to analyze the extent of inter-group variations following different normalization techniques. A higher *p*-value indicates a lesser disparity among the groups. It can be seen that the methods employed by DESeq2(ratio, poscounts) exhibited the highest level of stability (Fig. 6 B-D, Supplementary Fig. 15, Table 6, Supplementary Table 6). The minimal variation among the seven standardization methods was attributed to the high quality of the data (Fig. 6B, Supplementary Fig. 15). However, in the case of poor data quality, the two standardization methods of DESeq2 still ensure high-quality standardized output results (Fig. 6C).

**Table 6:** The results of Kruskal-Wallis rank sum test

*M.oryzae*			*V.dahliae*			*C.gloeosporioides*		
	chi_squared	p_value		chi_squared	p_value		chi_squared	p_value
poscounts	16.77	4.96E-3	poscounts	8.50	0.13	poscounts	27.50	4.56E-05
ratio	18.74	2.15E-3	RLE	8.56	0.13	ratio	63.04	2.86E-12
RLE	42.03	5.80E-08	ratio	8.70	0.12	RLE	675.60	9.24E-144
TMM	45.57	1.11E-08	TMMwsp	18.71	2.18E-3	TMMwsp	678.88	1.81E-144
UQ	47.11	5.40E-09	TMM	19.34	1.66E-3	TMM	694.46	7.74E-148
TMMwsp	49.00	2.22E-09	UQ	58.40	2.61E-11	UQ	1144.20	3.58E-245
none	608.53	2.90E-129	none	374.1	9.70E-79	none	2535.26	0

**Fig. 7:**
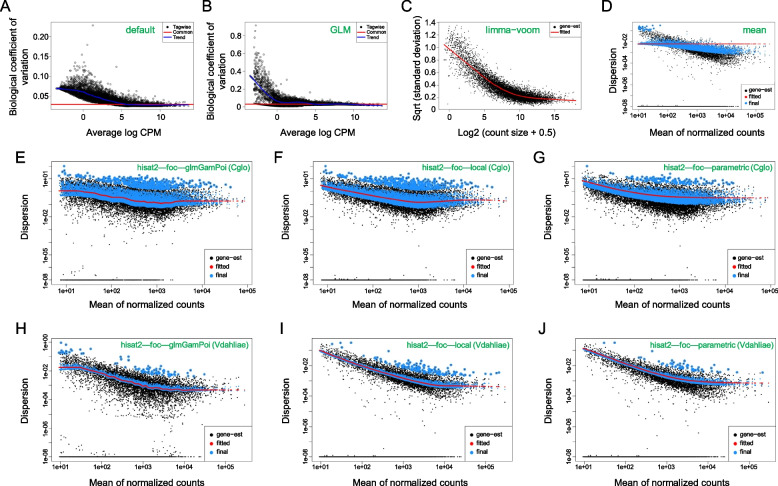
Visualization of the results of each fitting methods. **A-D** the performance of four methods using *V. dahliae* dataset. **E-J** a comparison was conducted on the three remaining fitting methods using datasets of varying data quality: one dataset with good data quality (Vdahliae, *V. dahliae*) and another with poor data quality (Cglo, *C. gloeosporioides*). If the model is well-fitted, the distribution of “Tagwise”(black) points will conform to the “Trend” (blue)curve in the edgeR package using the default or GLM method. In the limma-voom method, the “gene-est”(black) point will be fitted to the “fitted” (red)curve. When utilizing the DESeq2 package with the correlation (mean, glmGamPoi, local, parametric) method, the “final” (blue)points will exhibit a close distribution around the “fitted” (red)curve

Seven dispersion fitting methods were assessed, revealing that the mean method of the DESeq2 package was completely unsuitable for fungal data (Fig. 7D, Supplementary Fig. 16). Furthermore, the edgeR package (default, GLM) and limma-voom (voom) exhibited ordinary performance when utilized with the *V. dahliae* dataset, which was distinguished by its high data quality (Fig. 7A-C, Supplementary Fig. 16). Consequently, these four methods were excluded from subsequent comparative analyses.

Compared with the other three fitting methods, it was found that DESeq2 parametric fitting method demonstrated superior stability, with a high fitting degree in all data (Fig. 7E-J, Supplementary Fig. 16). The glmGamPoi fitting method in DESeq2 often had better performance when applied to high-quality data. However, it exhibited the longest running time compared to all other fitting methods, taking approximately 1 min and 30 s to complete, whereas alternative fitting methods required less than a second to run. Therefore, the most suitable method is parametric due to its stable performance. Furthermore, we compared the performance of various normalization and fitting methods across plant (*P. tomentosa*) and animal (*M. musculus*) datasets, revealing robustness across species for each method. Methods exhibiting superior performance on fungal data also demonstrate effective implementation in other species datasets (Supplementary Figs. 17, 18). The distinctions of various parameter combinations will be described in the Global analysis section.

### Global analysis

**Fig. 8:**
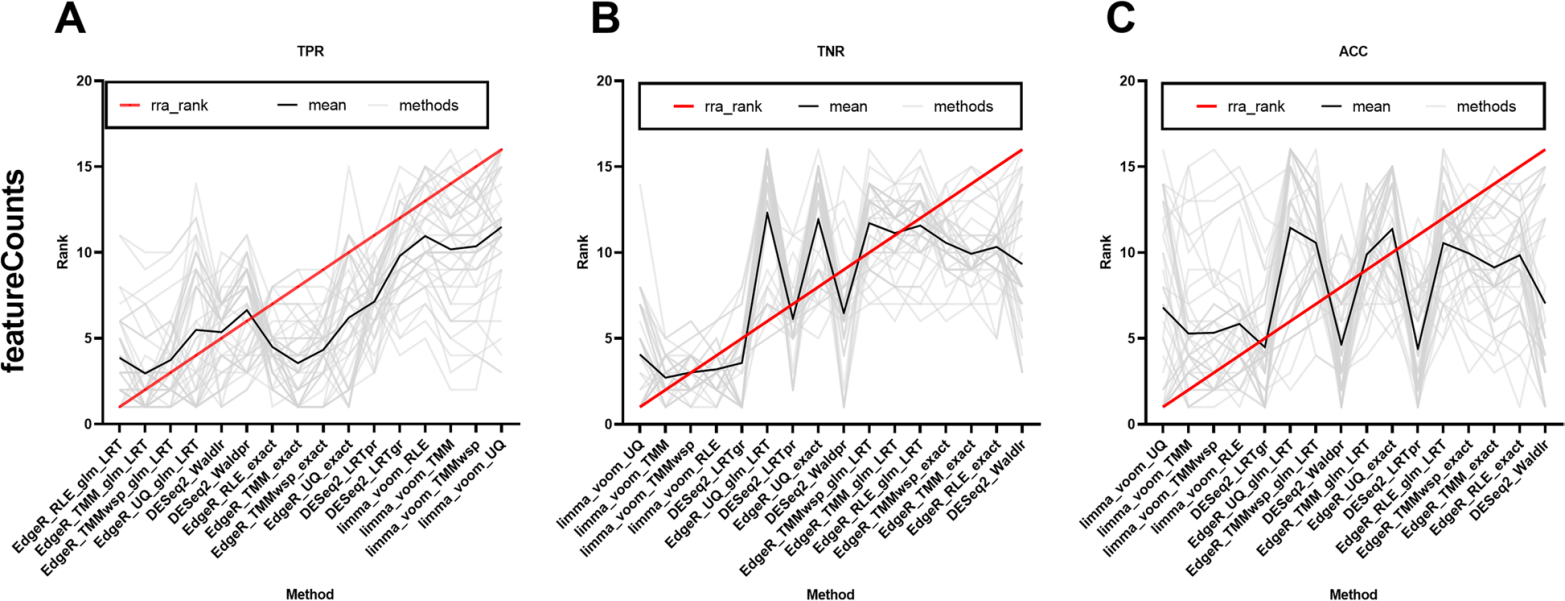
The TPR, TNR, ACC of each analysis workflow under featureCounts

After comparing a single software, the results of the entire process are further compared and analyzed. Here, we choose to calculate true positive rate (TPR), true nagative rate (TNR) and accuracy (ACC) under each method as the criteria for assessing the advantages and disadvantages of the method (Fig. 8). First, we performed an overall comparison of the 16 differential gene analysis combinations. Based on the Robust RankAggreg package [78], the comprehensive ranking of the five fungal datasets indicated that the combination involving the edgeR package consistently exhibited a higher TPR (Fig. 8A), limma-voom package-related combination usually had a higher TNR(Fig. 8B), while the limma-voom-related method generally showed a higher ACC(Fig. 8C).

During the experimental procedure, it was observed that in addition to the differences in the results of differential expression of genes, there was also a certain proportion of misjudgment in the results of differential expression, such as the identification of up-regulated genes in the validation data set as down-regulated genes. Through visualization, it is found that this phenomenon mainly exists in data with slightly poor sequencing quality, which once again proves the necessity of ensuring sequencing quality.

**Fig. 9:**
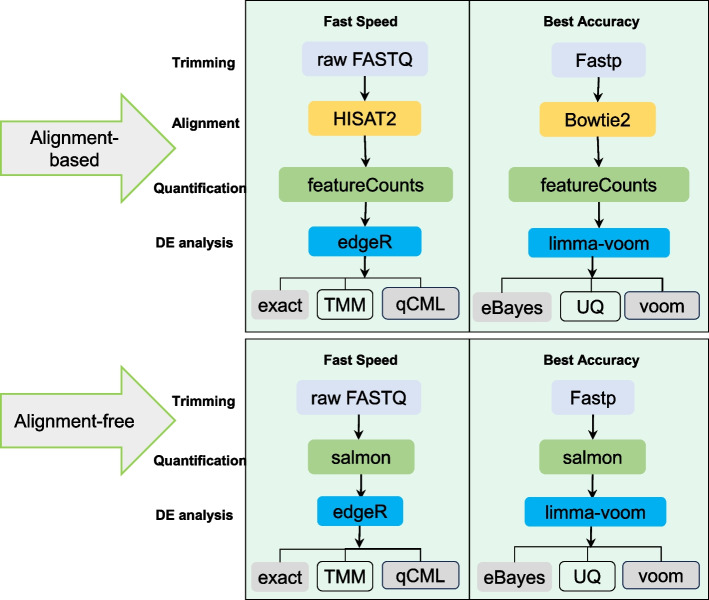
The optional workflow of RNA-seq data analysis

Among the upstream analysis methods, Bowtie2 demonstrated superior performance in the three fungal datasets, as indicated by higher values of true positive rate (TPR), true negative rate (TNR), and overall accuracy (ACC) (Additional file 1). This demonstrates that utilizing Bowtie2 can yield more precise outcomes. Based on the above comparison results, we propose a reference analysis process and divide it according to different needs (Fig. 9).

## Discussion

Through a comprehensive analysis of RNA-seq, considering different steps, we observed a significant impact of tool and computational method selection on the precision and runtime of the analysis. First, we compared each step separately.

During the quality control filtering process, if user want to quickly get the quality control report at the same time, they can consider using quality control filtering software combined with tools such as FastQC. However, Trim_Galore is not advisable due to suboptimal filtering results. The fastp is a good choice for researchers to start their analysis due to its rapid speed, consistent filtering outcomes, and moderate utilization of computational resources.

The experimental results affirm that judicious trimming leads to the most optimal enhancement in data quality and has the highest comparison rate improvement degree. This conclusion aligns with findings from previous research [26]. Additionally, we proposed two selection criteria for trimming parameters, referred to as FOC and TES respectively, which can help users decide the parameters according to the characteristics of the data itself. Upon comparing the results with subsequent analysis steps, it was observed that for RNA-seq data of satisfactory quality, the trimming and filtering step had minimal impact on the results of subsequent quantification and differential expression analysis. This finding aligned well with the conclusion drawn by Liao et al. [79]. Therefore, for fungal trimming and filtering step, we recommend considering the quality of the obtained data and the research objectives. If the data itself is of good quality and the analysis goal is focused on differential gene selection, it may be feasible to only perform adapter trimming without other treatment.

During the alignment step, as the ENCODE-recommended RNA-seq alignment tool, STAR is widely used in comprehensive workflows [31, 80, 81]. In this study, despite STAR's excellent mapping rate, considering the runtime and resource consumption, HISAT2 emerged as the superior choice. Bowtie2 exhibited the lowest mapping rate across all datasets, possibly due to its design for DNA alignment and its relatively lower accuracy in handling intron sizes. However, when assessing the accuracy of DE results obtained by different alignment methods, Bowtie2 showed superior performance. Since this study focused on differential gene selection results, default parameters were employed for the alignment step. However, previous studies have shown that alignment software often yields suboptimal results when using default parameters, and parameter optimization can lead to improved outcomes. MIN_MAP_LENGTH, SPLICE_MISMATCHES, and APPEND_MISMATCHES were identified as the three parameters with the greatest impact on the performance of HISAT2. For STAR, increasing NUM_FILTER_MISMATCHES while retaining the default value of END_ALIGNMENT_TYPE can enhance the results [28].

During the quantification step, by examining the top 30% of processing pipelines based on evaluation metrics in each fungal data, it was found that over 50% of the processes utilized featureCounts as the quantification tool, followed by RSEM. This indicates that the differential gene analysis results derived from featureCounts exhibited optimal performance among the three quantification tools. Although Salmon provided the fastest running speed, its overall performance was significantly weaker compared to the other two alignment-based tools. This suggests that alignment-based tools are recommended for quantification in fungal data analysis. Additionally, it was observed that under the same thread number, the analysis time for combining featureCounts with STAR/Bowtie2 was comparable to the analysis time for using RSEM with STAR/Bowtie2. Therefore, if ease of use is a consideration, RSEM is recommended for analysis as it only requires learning one software for both alignment and quantification steps.

During the DE analysis step, a comparison was made both normalization methods and fitting methods. It was observed that different methods exhibited significant differences in robustness. DESeq2's two normalization methods showed strong robustness, yielding good normalization effects regardless of data quality. In contrast to previous studies, in terms of the performance of normalization, TMM did not always have the most stable effect in fungal data [82, 83]. Overall, the results were consistent with previous research [23, 84, 85], edgeR using the TMM method demonstrated a higher true positive rate (TPR).

When it comes to the 16 differential gene analysis methods after parameter combination, there are great differences among these methods. To comprehensively and intuitively evaluate the performance of different approaches in analyzing differential expression, we utilized the Robust RankAggreg package to rank the assessment metrics, considering the incorporation of multiple combinatorial procedures (Fig. 8, Supplementary Fig. 19). After comparing different workflows based on evaluation metrics, we observed that the choice of alignment software significantly influenced the results of differential expression (DE) analysis. When employing the identical differential analysis method on the same data, different alignment methods can lead to differences of approximately 0.04–0.2 in the evaluation metrics (Additional file 2). Similar situations were noted during the quantification step, where alignment-based methods outperformed alignment-free methods overall, but the distinctions among different alignment-based methods were relatively minor. Furthermore, substantial variations were observed in the evaluation metrics when different combinations of differential gene analysis methods were applied under the identical upstream processing workflow.

Considering the diverse needs among different populations in conducting fungal RNA-seq data analysis, we have outlined a reference workflow (Fig. 9) that takes into account both accuracy and efficiency. This workflow is divided into two major categories: alignment-based and alignment-free. We made this division because, although alignment-based methods generally outperform alignment-free methods, our results also revealed cases where the latter outperformed the former. Furthermore, considering its rapid analysis speed, the alignment-free method can serve as a preliminary exploration tool for the data. As indicated by previous studies [16, 22, 86], combination of DE results from multiple workflows can yield more accurate DEGs. We also suggest that for the purpose of data analysis, several processes be selected for multiple tests based on the evaluation of each step and the global process in this study.

In addition to analyzing workflows tailored for differential gene selection, our research also conducted a simple comparison of tools for alternative splicing analysis. For AS analysis in the fungal RNA-seq data, the results indicates that the pipeline combining Bowtie2 is not incapable of performing AS analysis. Instead, it exhibits abnormal performance on fungal datasets. Therefore, for fungal datasets, we recommend combining the other two alignment software for AS analysis pipelines. The results using the combination of rMATS and SpliceWiz yielded superior results, demonstrating a more comprehensive types and numbers of events.

## Conclusions

In conclusion, a thorough evaluation was conducted on the software utilized at each step of the analysis. A comparative analysis was conducted to assess the strengths and weaknesses of the software. Additionally, we evaluated the quality of the analysis results generated by various software when utilized with plant pathogenic fungal data. Instead of providing a best practice for RNA-Seq analysis, we presented a definitive reference workflow and identified potential differences that may arise due to the methods selected. This is because a process that is optimal for certain data may not exhibit the same level of performance when applied to different data sets. Based on our study, researchers can enhance the efficiency and accuracy of selecting appropriate analytical tools for the analysis of RNA-seq data.

## Materials and methods

### Data sets and code

Supplementary Table 7 summarizes the data sets used in this study [5, 41, 87–91]. All the analysis scripts are offered in the Additional file 3, Additional file 4 and Additional file 5.

### Environment

The RNA-seq tools utilized in this study, along with their corresponding versions, are specified in Supplementary Table 8. The computational analyses were performed on a CentOS server with a 48-core CPU, 96 threads, and 32GB memory.

### Filtering and trimming evaluation

Quality control (QC) reports were used to identify important base positions in the ATCG base proportion curves within the sequences, guiding the base trimming parameters. The curve represents the proportions of four different bases present at various sites, allowing detection of AT and GC separation phenomena. According to the author's description on the official website of FastQC, ideally, the distribution of the A, T, C, and G bases should be close to and parallel with each other. However, due to inevitable errors during library construction, such as the sequencing instrument not stabilizing in the initial bases or inherent biases in primers, fluctuations in base distribution can occur at the 5' end of sequencing data. We define this fluctuating and imbalanced state of base distribution as chaos. Specifically, our focus was on two significant positions (Supplementary Fig. 20, 21): the point at which the ATCG base proportion curves out of chaos for the first time (FOC), indicating where the fluctuations in the curve disappear (Supplementary Fig. 21A), and the point at which they reach an equilibrium state (TES), representing the position where the curve fluctuations diminish and approach parallelism (Supplementary Fig. 21B).These two key thresholds were determined with the help of the MultiQC tool, which provides html reports that allow users to automatically browse the corresponding base positions and their balance ratio distribution data when they move the mouse.

Throughout the subsequent discussions in this paper, the terms "raw," "FOC," and "TES" are utilized as descriptors for datasets under distinct processing conditions. The filtering parameter was consistently set at 25. Notably, Trim_Galore integrates Cutadapt and FastQC, enabling the direct generation of QC reports. When evaluating the computational efficiency of the two tools, the running time of fastp is a combination of its own execution time and the time required to utilize FastQC to generate the QC report.

### Read mapping evaluation

The datasets generated under three distinct processing conditions (raw, FOC, TES) were aligned to the reference genome utilizing the most widely employed tools, namely HISAT2, STAR, and Bowtie2. The quantification of mapping instances for each sequencing read when employing STAR and HISAT2 was ascertained by referencing the NH tag within the alignment file. In the case of Bowtie2, a sequencing read is deemed uniquely mapped when the AS tag and its corresponding value are both non-empty, and there is an absence of the XS tag in the alignment file. Should the value associated with the AS tag be null, the read is classified as unaligned. Conversely, if the value is populated, the read is categorized as multiply aligned. The count of soft-clipped bases was derived from the alignment CIGAR string, whereas the detection of mismatches relied on the NM tag. Building upon previous research [21], we added the analysis of Bowtie2 into the code.

### Quantification evaluation

To evaluate the quantitative results, we selected several prominent software packages (featureCounts, RSEM, and Salmon) for a thorough comparison. Certain quantitative software is capable of accepting the output generated by specific alignment software, while others have the ability to directly process the raw fastq file. The distinctive combinations are illustrated in Fig. 1.

To explore the impact of upstream processes on quantitative outcomes, we computed the correlation between results obtained from identical samples but processed through distinct upstream processing workflows. Moreover, in order to investigate the sources of quantitative variances across different analytical scenarios, we ranked the quantitative results in a descending order and segmented them according to percentiles (1, 10, 25, 50, 75, 90, 95). Across different percentile divisions, we compared the correlation between quantitative outcomes obtained through distinct trimming and filtering measures in the processing workflow, as well as the correlation between quantitative results derived from diverse alignment software utilized in the processing workflow.

### Differential gene expression analysis evaluation

After estimating gene and transcript expression levels, researchers utilize statistical methods to identify variations in expression levels among different experimental groups [10]. Various approaches are available to accurately detect differentially expressed genes, which can be divided into two types: parametric and non-parametric. The classification of analytical methods adopts or describes the use of some statistical distribution of parameters to infer DEGs, as well as tools that either partially or entirely rely on this category of statistical distribution are categorized as parametric [33].

In this study, a comprehensive comparative analysis was performed using three widely adopted count-based tools for the analysis of differential gene expression (DE) in RNA-seq data: DESeq2, edgeR, and limma, which are extensively utilized in the field of RNA-seq analysis. Initially, we evaluated a single normalization method and a fitting method independently. The Kruskal–Wallis rank sum test was used to assess the magnitude of differences in data between samples based on standardized results. Subsequently, a total of 16 unique analysis combinations were created through the modification of normalization parameters, hypothesis testing parameters, and fitting parameters. These combinations were systematically employed to conduct comparative experiments during the stage of analyzing differential gene expression, as illustrated in Fig. 1.

Real experimental data were utilized to perform analyses involving 16 different methodological combinations. The outcomes of these analyses were then overlapped to establish a validation data, which was employed for evaluating the performance of the simulated data. To simulate data, we employed the data simulation tools, followed by the application of differential gene analysis techniques on the generated simulated data. Subsequently, the results obtained from the simulated data were compared with the validation data derived from real experimental data. The target set of DE genes was defined as those exhibiting a known absolute log2-fold change exceeding 1, accompanied by a corresponding *p*-value below 0.001. Various evaluation metrics, such as True Positive Rate (TPR), True Negative Rate (TNR), and overall Accuracy (ACC), were computed for each method, providing a comprehensive assessment of the differential gene analysis approaches employed in this study. Robust RankAggreg package was applied to rank the assessment metrics.

$$True Positive Rate (TPR) = TP / (TP + FN)$$

$$True Negative Rate (TNR) = TN / (TN + FP)$$

$$Accuracy (ACC) = (TP + TN) / (TP + TN + FP + FN)$$

TP: The count of genes that have been correctly identified as DE genes in the simulated dataset.

TN: The count of genes correctly identified as non-DE genes in the simulated dataset.

FP: The count of genes erroneously labeled as DE genes in the simulated dataset.

FN: The count of genes erroneously labeled as non-DE genes in the simulated dataset.

### Alternative splicing evaluation

Considering that the aim of this research is to offer a user-friendly analysis process for laboratory researchers with limited computer skills, the ease of use and practicability should also be considered when selecting the software. While the majority of contemporary methodologies can assess differential splicing across various sample groups, some of the earlier tools are limited to comparing differences between only two individual samples. MISO is a software that exhibits limitations in its utility for many studies. Consequently, three event-based AS tools, rMATS, SpliceWiz, and Whippet, were ultimately chosen as the comparative entities in this section.

Due to the absence of laboratory-verified alternative splicing data, this section focuses on comparing the disparities in AS analysis among various software tools based on several factors: the absolute number of AS events identified through analysis, the overlap of different AS events, and the running time of the software. To provide a more comprehensive comparison of software efficiency, it is essential to consider the time expended in the alignment phase when calculating the overall runtime of the rMATS and SpliceWiz software.

When using simulation tools [92], since the probabilities of different AS events vary among species, we used rMATS- detected AS event proportions from real *V. dahliae* RNA-Seq data aligned by STAR as input parameters to match AS event distribution in simulated data with real data. The simulated data obtained from this tool comprised multiple sequence files and a validation dataset representing the AS events generated during data simulation. After analyzing the simulated data using different software, we compared their results with the validation dataset to obtain performance metrics for different tools. Precision and recall were calculated using the following formulas:

1 $$Precision=TP\_event/find\_by\_tool\_event$$

2 $$Recall= TP\_event/validation\_event$$

TP_event counts AS events consistent with the validation dataset, find_by_tool_event tallies events identified by each software tool, and validation_event denotes the total events in the validation dataset. It is noteworthy that when calculating metrics for different event types, find_by_tool_event and validation_event represents the total number of corresponding event types identified by each tool and corresponding event types in the validation dataset, respectively.

In addition, we compared the splice junction output of STAR and rMATS results in the alternative splicing events between control group and treatment group. Taking RI events in the simulated Verticillium dahliae dataset as an example, we extracted IR events from the SJ.out.tab file, in conjunction with the GTF annotation file.

### Supplementary Information

**Supplementary Material 1.**

**Supplementary Material 2.**

**Supplementary Material 3.**

**Supplementary Material 4.**

**Supplementary Material 5.**

**Supplementary Material 6.**

**Supplementary Material 7.**

## Data Availability

All the datasets used can be found in European Nucleotide Archive(https://www.ebi.ac.uk/) with the accession number listed in the Supplementary Table 7, except for the V. dahliae dataset, which can be found in NCBI Sequence Read Archive (SRA) database (https://www.ncbi.nlm.nih.gov/sra) with the accession number PRJNA1109783. Additionally, all the analysis scripts used in this study were provided within supplementary information files. M. oryzae https://www.ebi.ac.uk/ena/browser/view/PRJNA523930 C. gloeosporioides https://www.ebi.ac.uk/ena/browser/view/PRJNA391239 V. dahliae https://www.ncbi.nlm.nih.gov/sra/?term=PRJNA1109783 P. tomentosa https://www.ebi.ac.uk/ena/browser/view/PRJNA561520 M. musculus https://www.ebi.ac.uk/ena/browser/view/PRJNA886709 U. maydis https://www.ebi.ac.uk/ena/browser/view/PRJNA998905 R. stolonifer https://www.ebi.ac.uk/ena/browser/view/PRJNA940265
