# Supplementary material for: A comprehensive workflow for optimizing RNA-seq data analysis
Source: BMC Genomics. 2024 Jun 24;25:631. doi: 10.1186/s12864-024-10414-y (PMC11197194; doi:10.1186/s12864-024-10414-y)
Supplement: Supplementary file 1 — Supplementary Material 1. [file 12864_2024_10414_MOESM1_ESM.docx]

**Supplementary Fig. 1** Phylogenetic tree of fungi. The red rectangle in the figure represents the category to which the data involved in this study belong.

**Supplementary Fig. 2** Phylogenetic tree of fungi.The red marker points to the location of the fungal species used in this study in the evolutionary tree.


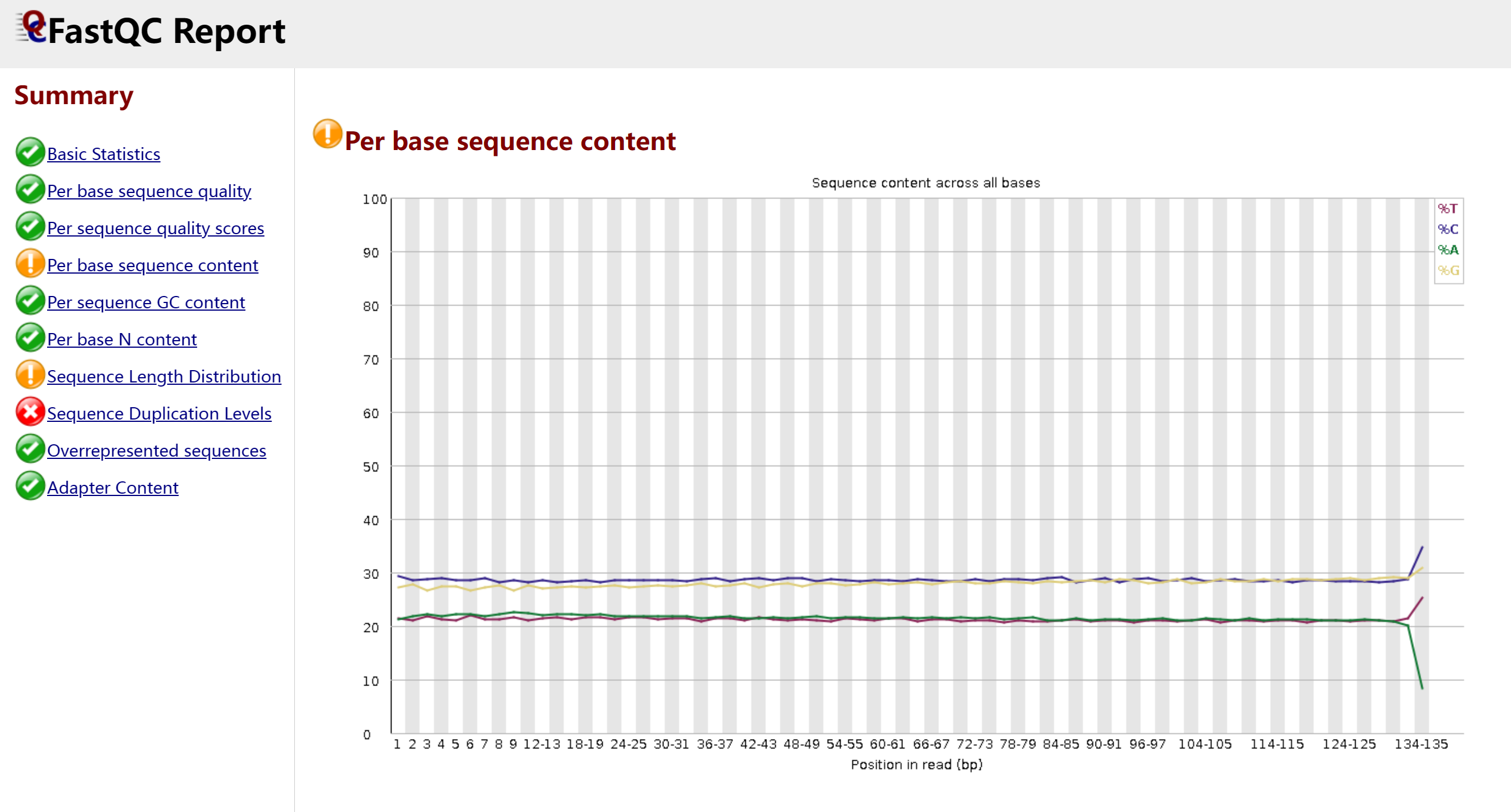


**Supplementary Fig. 3** The tail bases of the data processed with Trim_Galore.


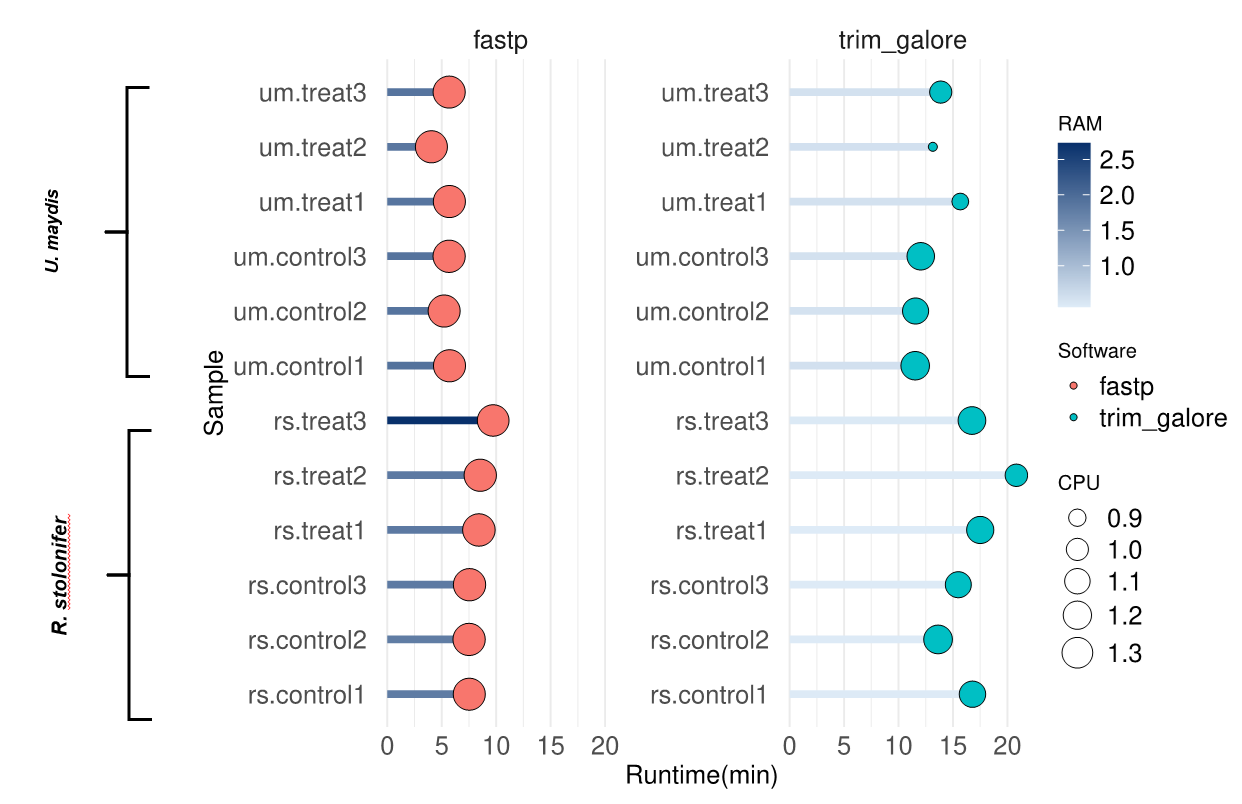


**Supplementary Fig. 4** Runtime, CPU and RAM occupancy of different tools. Comparison between Trim_galore and fastp.


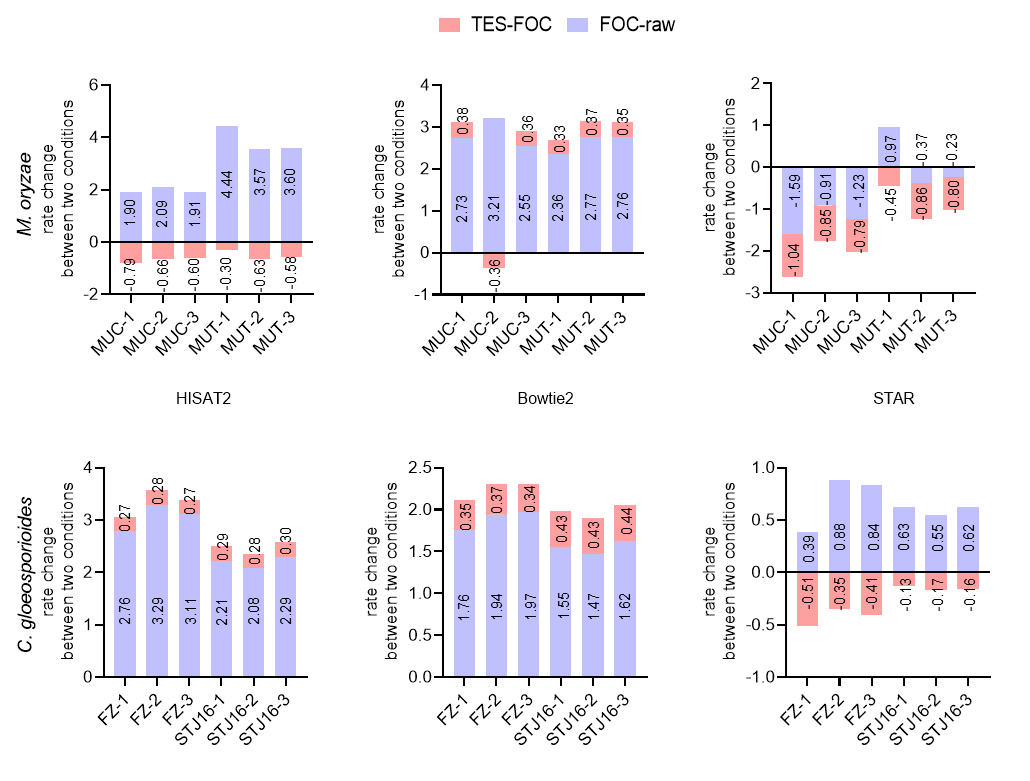


**Supplementary Fig. 5** The bar chart illustrates changes in alignment rates.


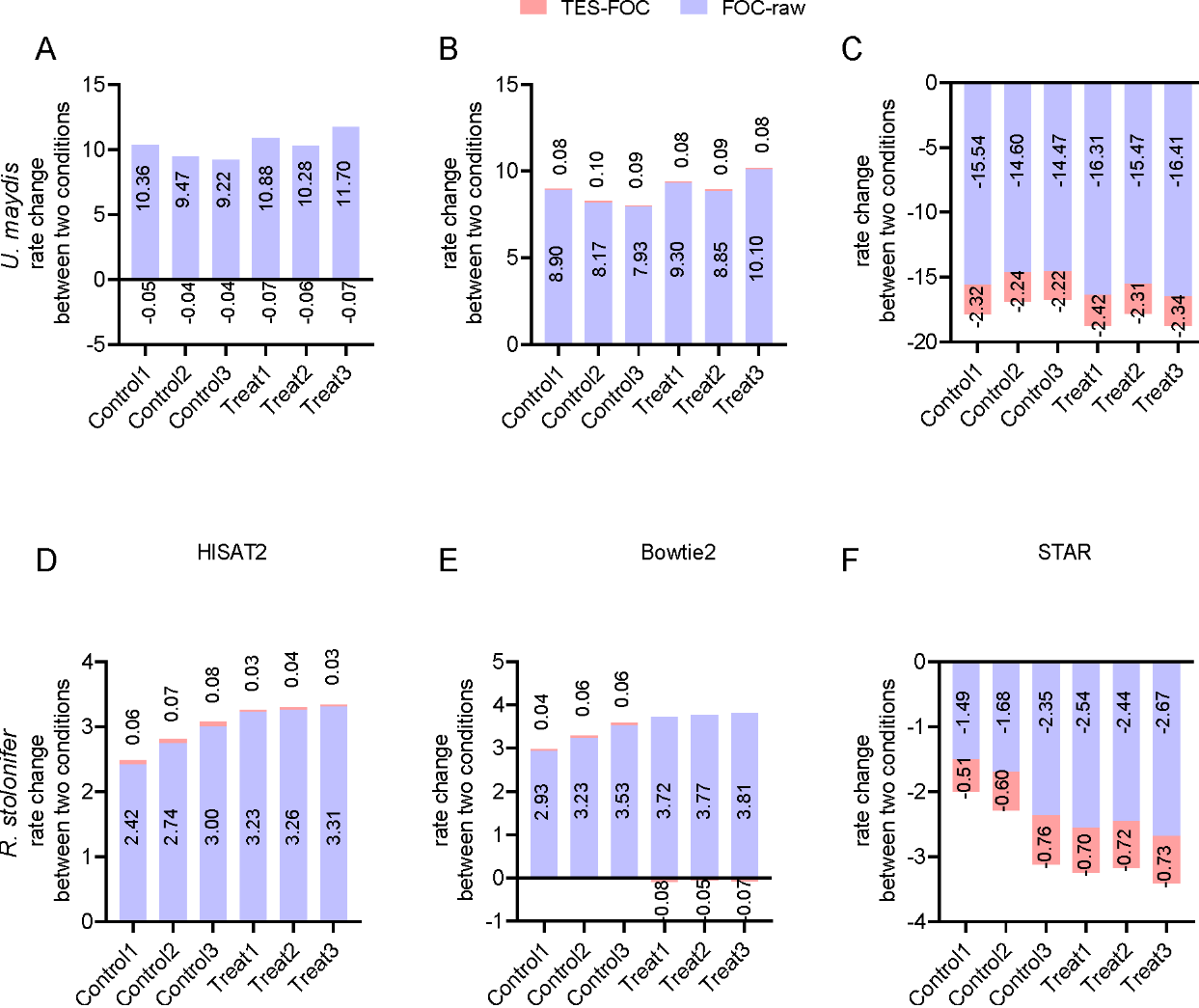


**Supplementary Fig. 6** The bar chart illustrates changes in alignment rates.


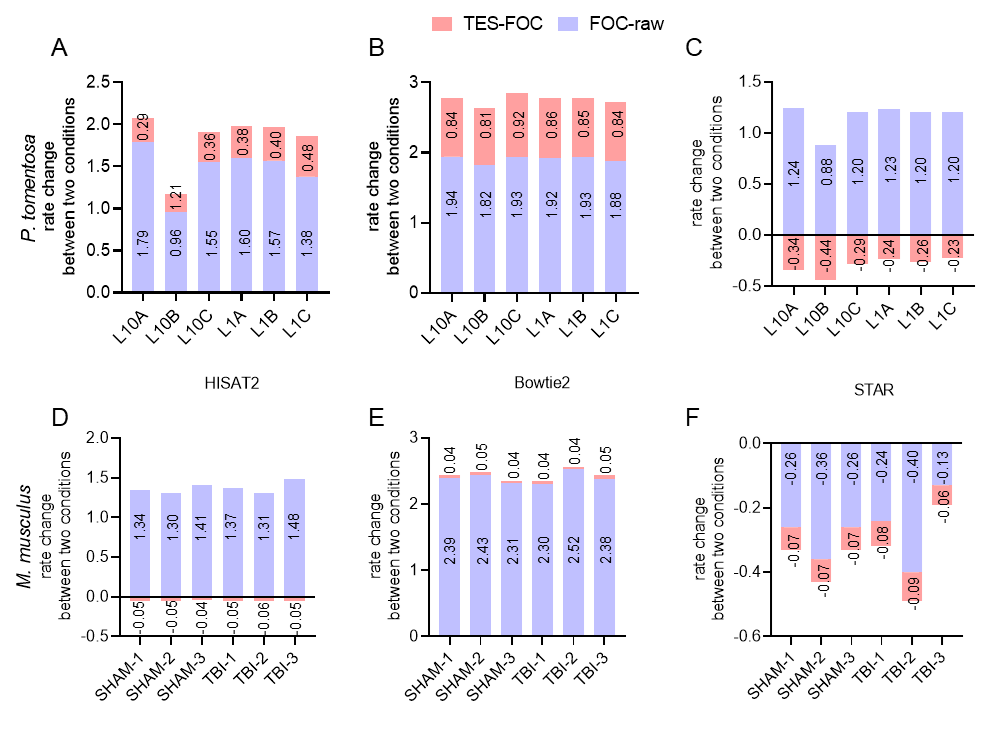


**Supplementary Fig. 7** The bar chart illustrates changes in alignment rates.


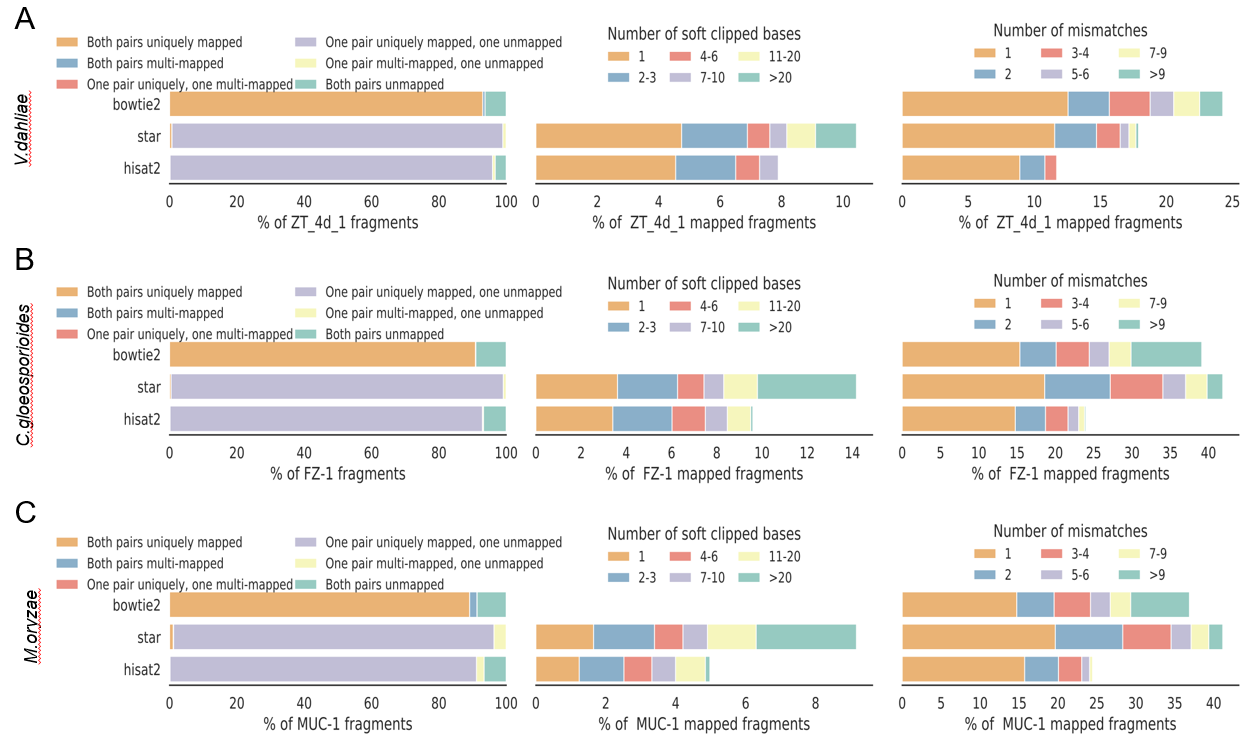


**Supplementary Fig. 8** Performance of different alignment softwares. Read mapping analysis: distribution of mapping status of sequenced fragments (left) (mapping status for paired-end reads，the distribution reflects percentage of uniquely mapped (blue), multi-mapped (orange), and unmapped (red) single-end reads are shown), distribution of number of soft-clipped bases in mapped fragments (middle), distribution of the number of mismatches in mapped fragments (right).a, b and c correspond to the comparison of MUC-1 in three kinds of M.oryzae samples under trimming and filtering treatment, respectively.


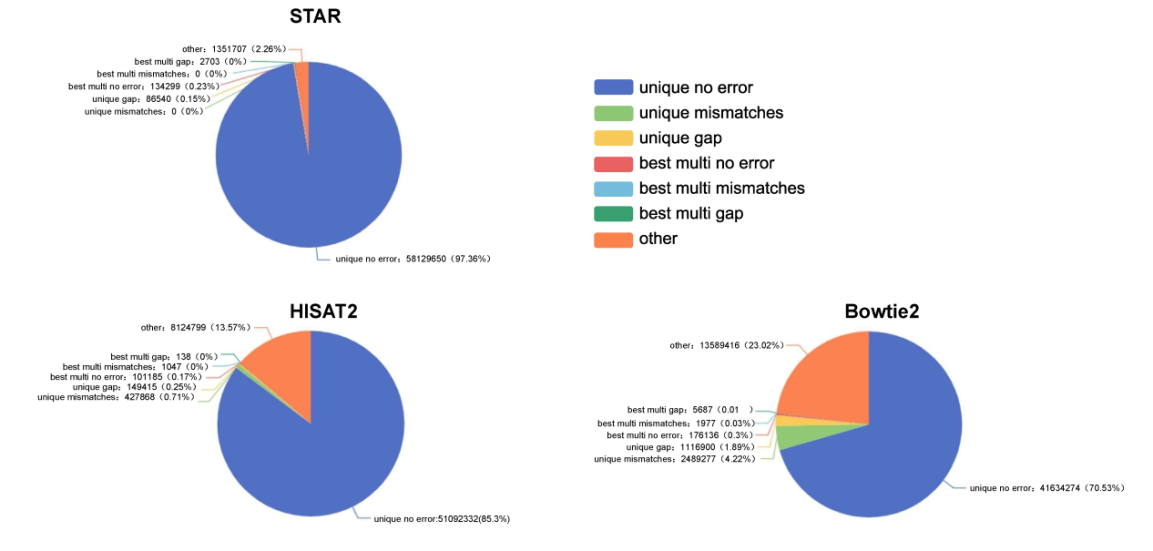


**Supplementary Fig. 9** The alignment details in three aligners

R. stolonifer

A

B

U. maydis

**Supplementary Fig. 10** Runtime, CPU and RAM occupancy of different tools. Comparison between different aligners under the same running threads.


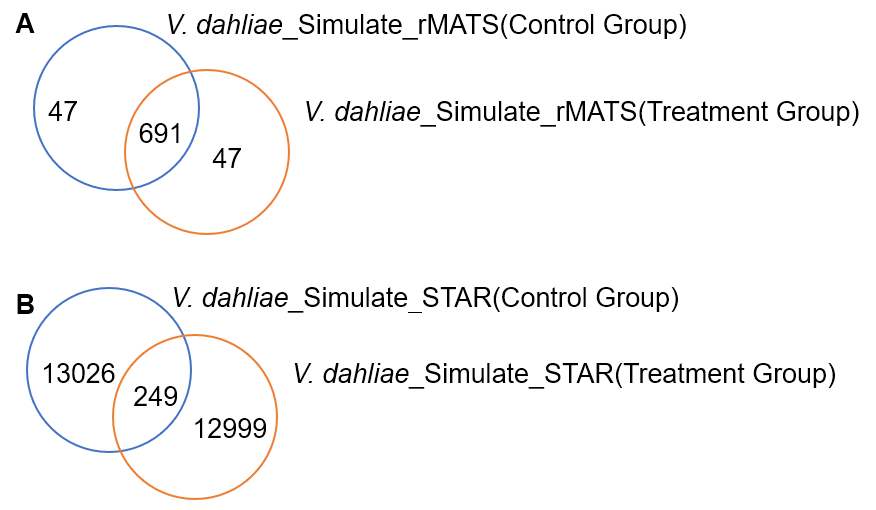


**Supplementary Fig. 11** The visualization of alternative splicing events between Control group and Treatment Group in *V. dahliae* via rMATS and STAR software.


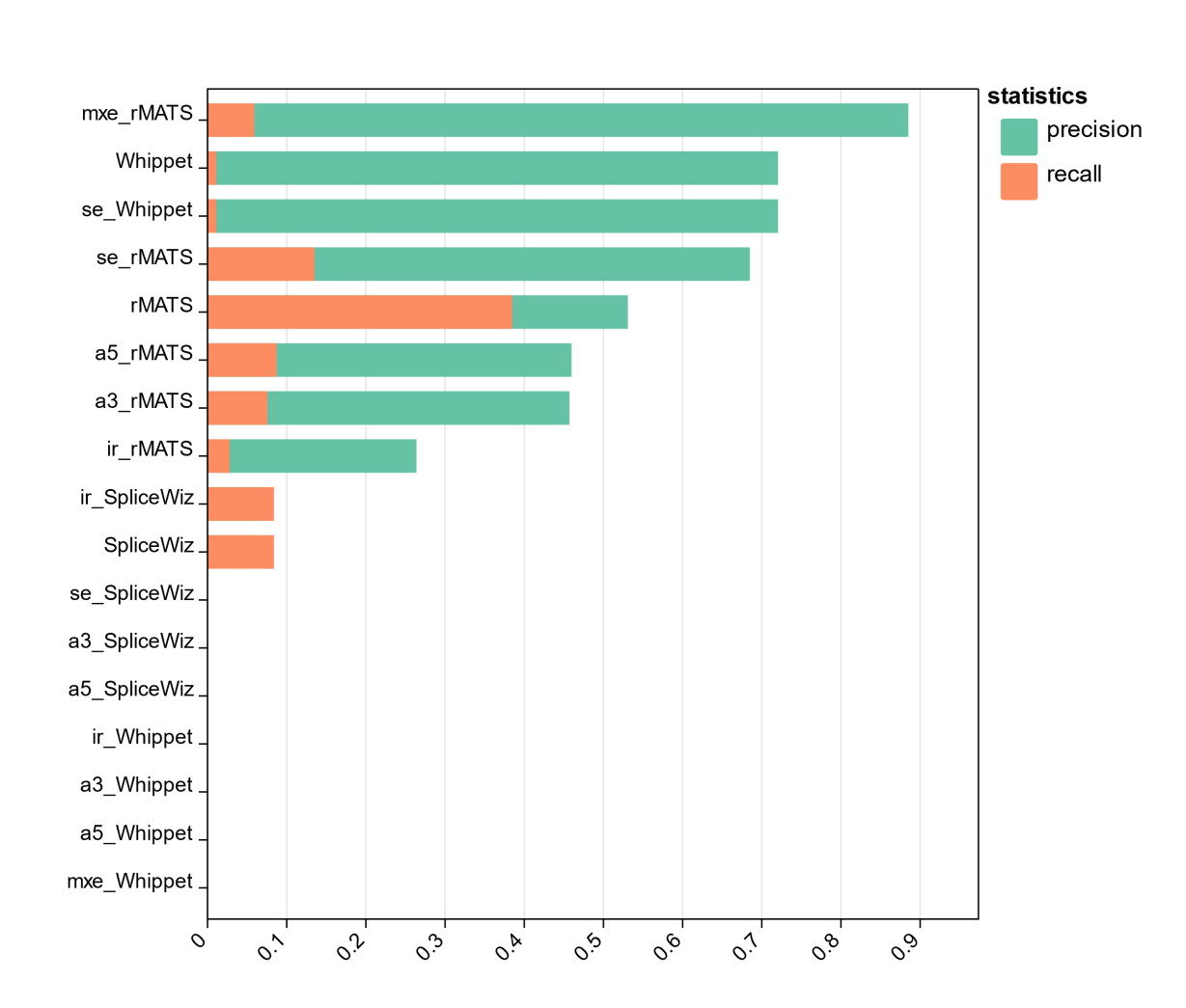


**Supplementary Fig. 12** The recall and precision of various AS tools


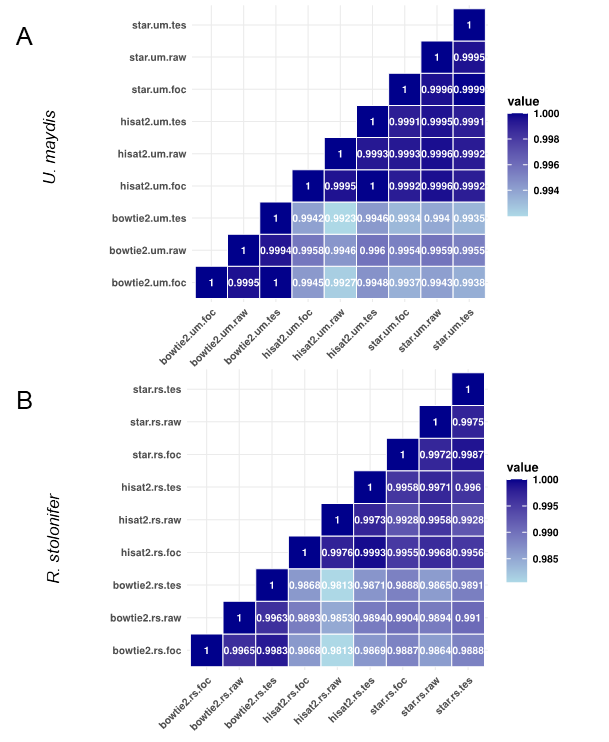


**Supplementary Fig. 13** Calculations of different quantification results. Heatmap of different schemes using feature counts as quantification tool based on the Spearman rank correlation of their log expressions in (**A**)*U. maydis* and (**B**) *R. stolonifer*

R. stolonifer

A

C

U. maydis

B

U. maydis

R. stolonifer

D

**Supplementary Fig. 14**  Correlation between the quantification results of different quantification tools(**A**, *U. maydis*;**B**, *R. stolonifer*).Correlation between the quantification results of different trimming and filtering treatment, both using raw data as benchmark data(**C**, *U. maydis*;**D**, *R. stolonifer*)


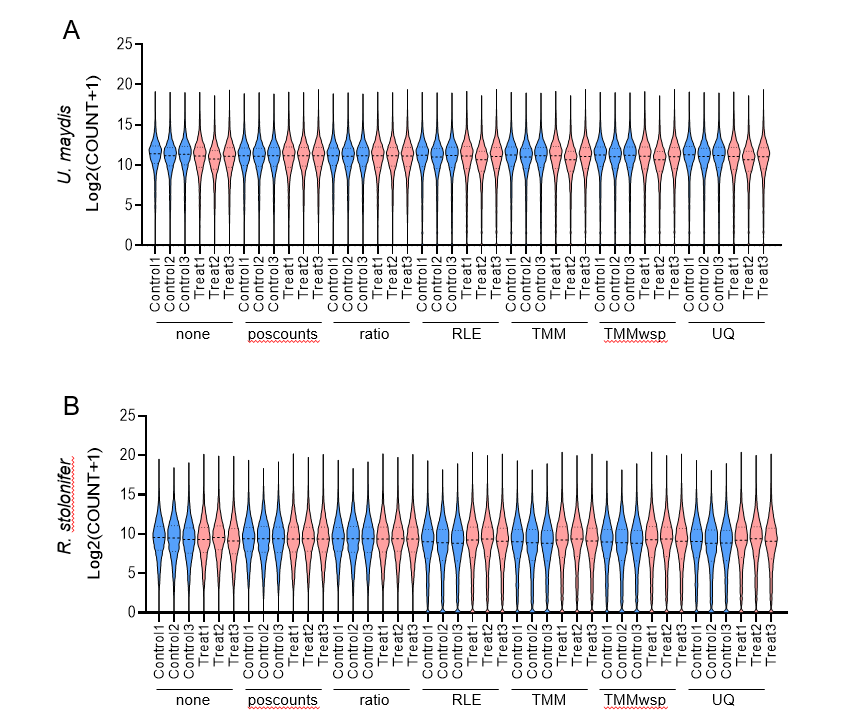


**Supplementary Fig. 15** The data distribution of different normalization methods in (**A**) *U. maydis*

and (**B**) R. stolonifer


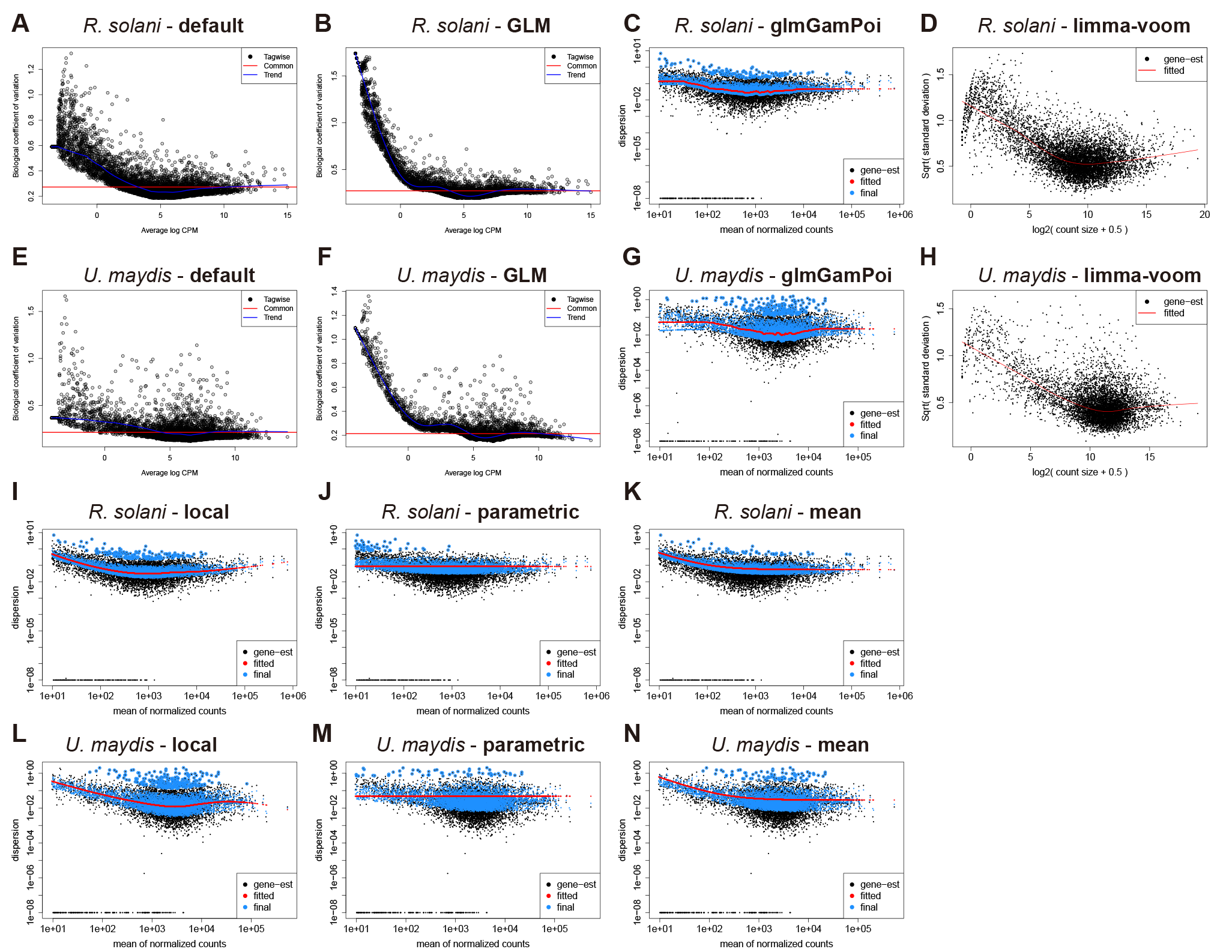


**Supplementary Fig. 16** The visualization of the results of each fitting methods of (**A-D, I-K**)*R. stolonifer* and (**E-H, L-N**)*U. maydis*

.


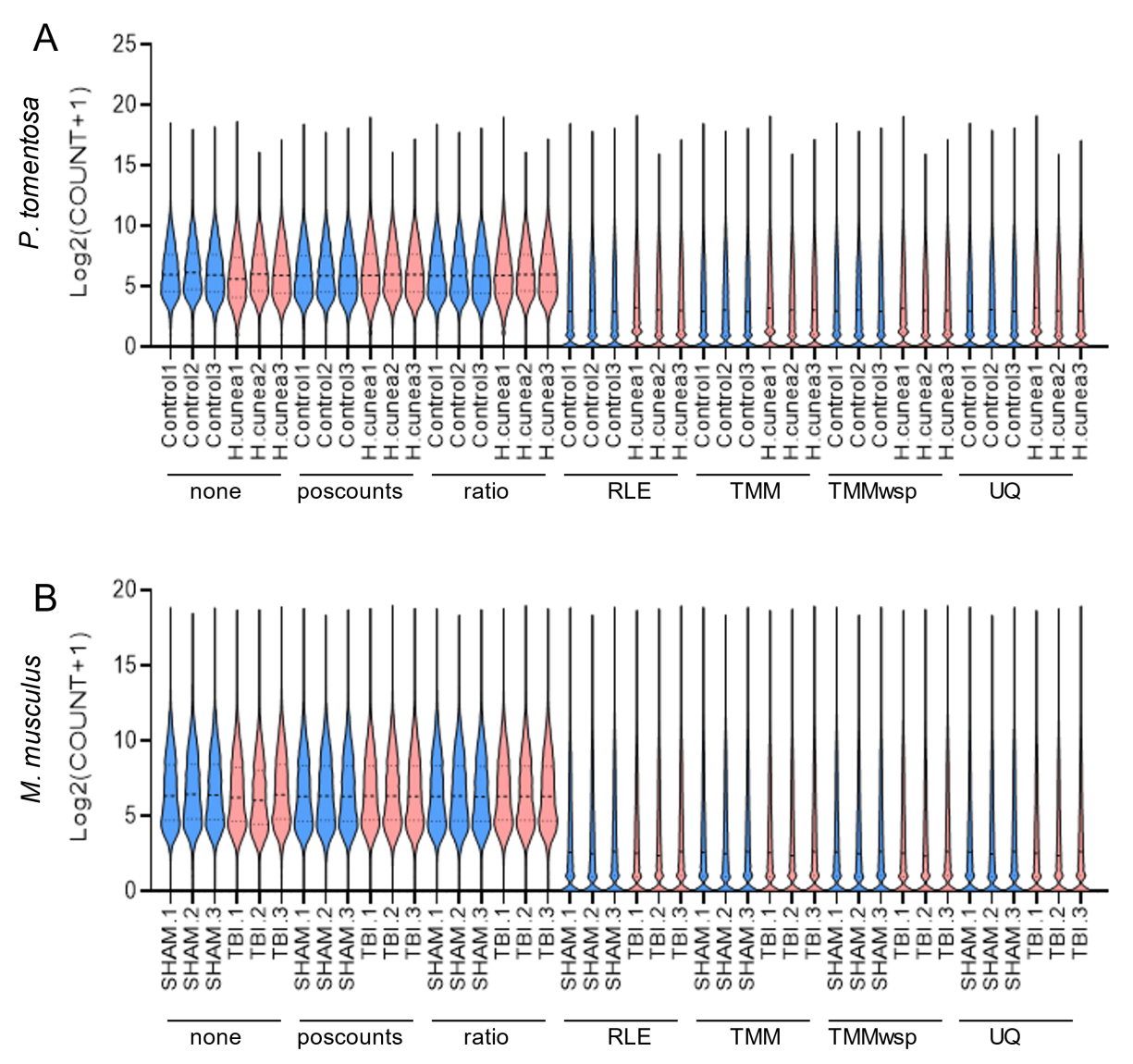


**Supplementary Fig. 17** The data distribution of different normalization methods in (**A**) *P. tomentosa* and (**B**) *M. musculus*


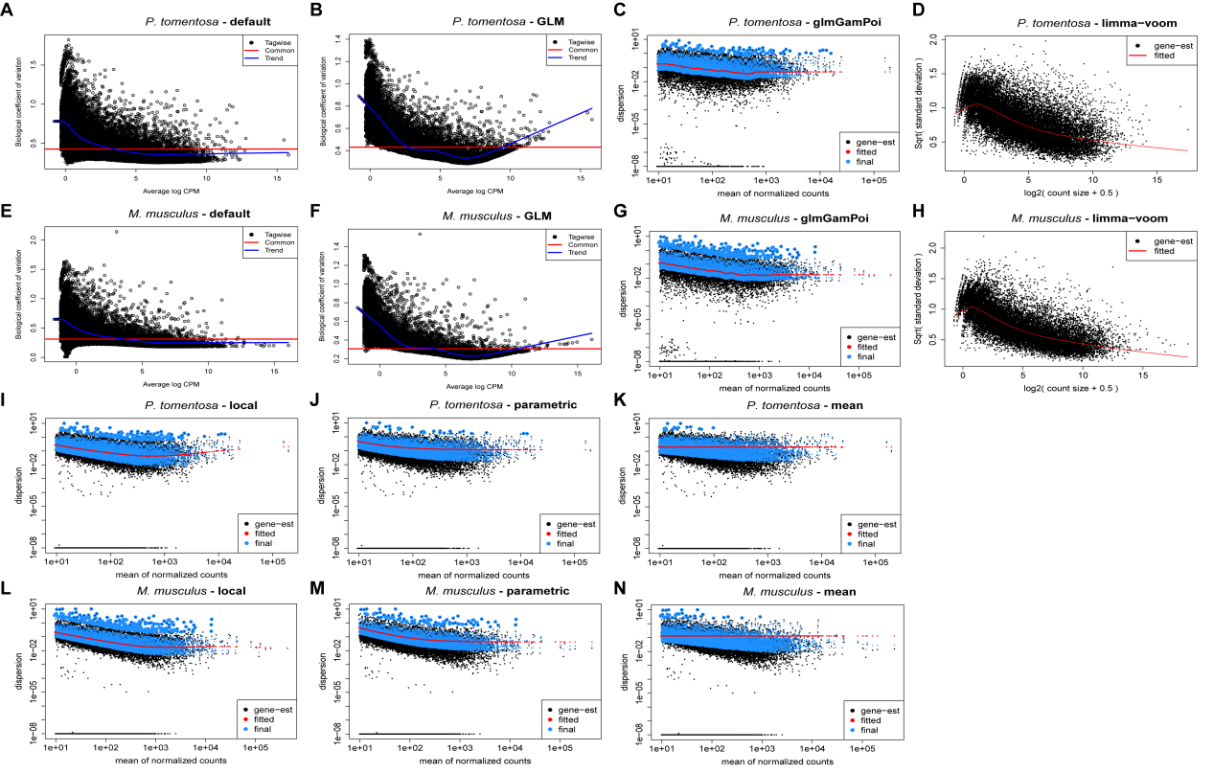


**Supplementary Fig. 18** The visualization of the results of each fitting methods of (**A**)*P. tomentosa* and (**B**)*M. musculus*

**Supplementary Fig. 19** The TPR、TNR、ACC of each analysis workflow under different quantification tools.


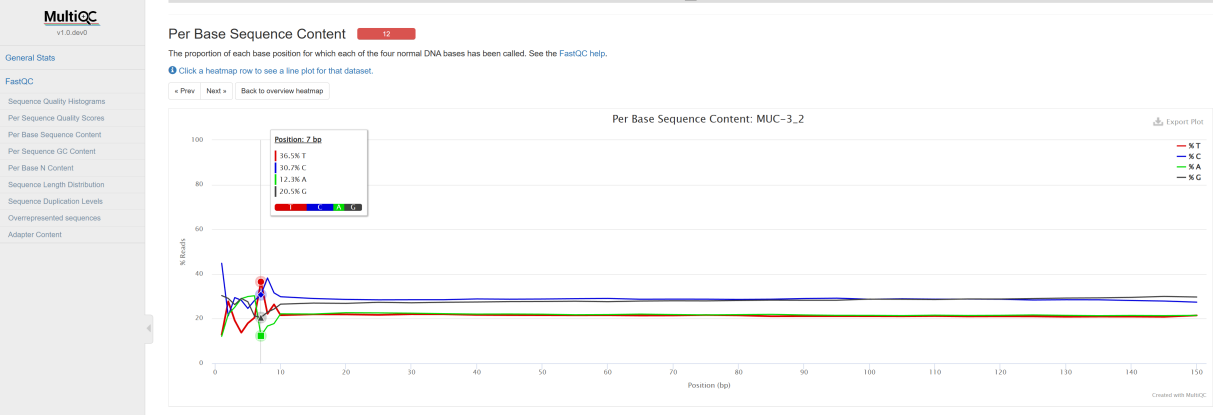


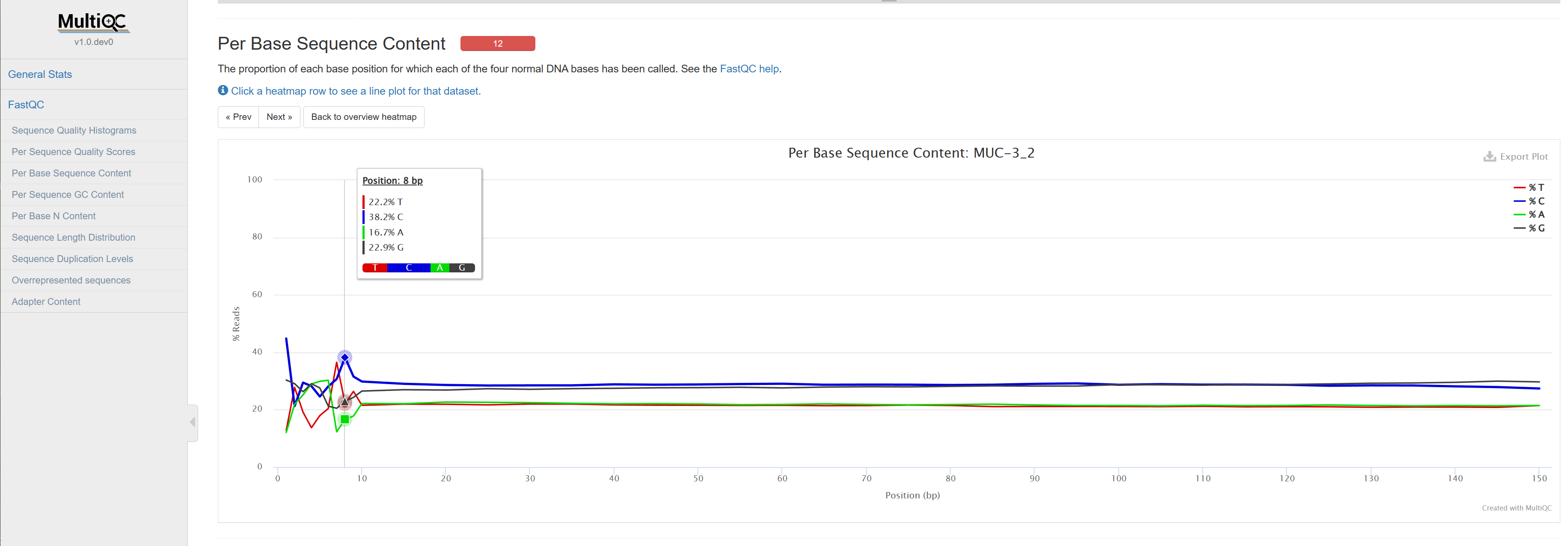


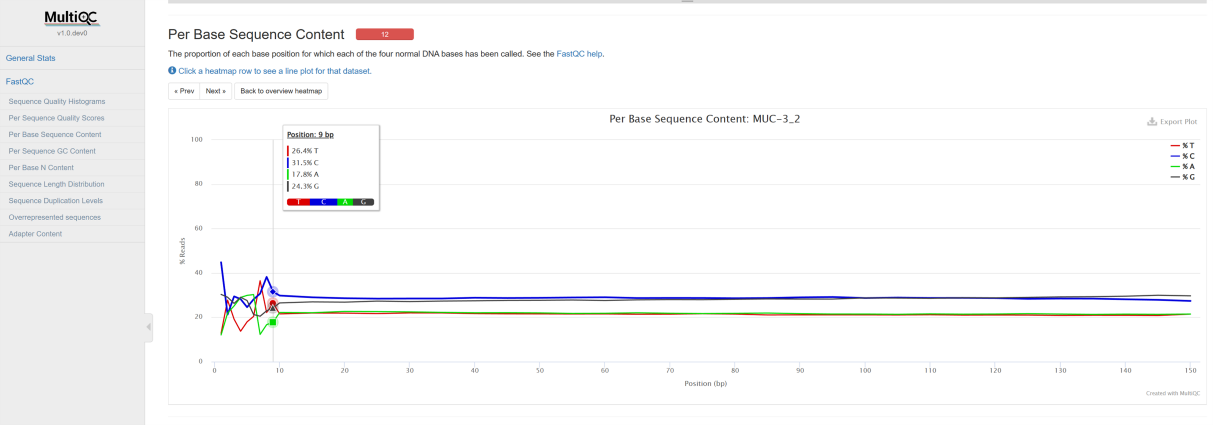


**Supplementary Fig. 20** The information of ATCG base proportion curves in different base location.


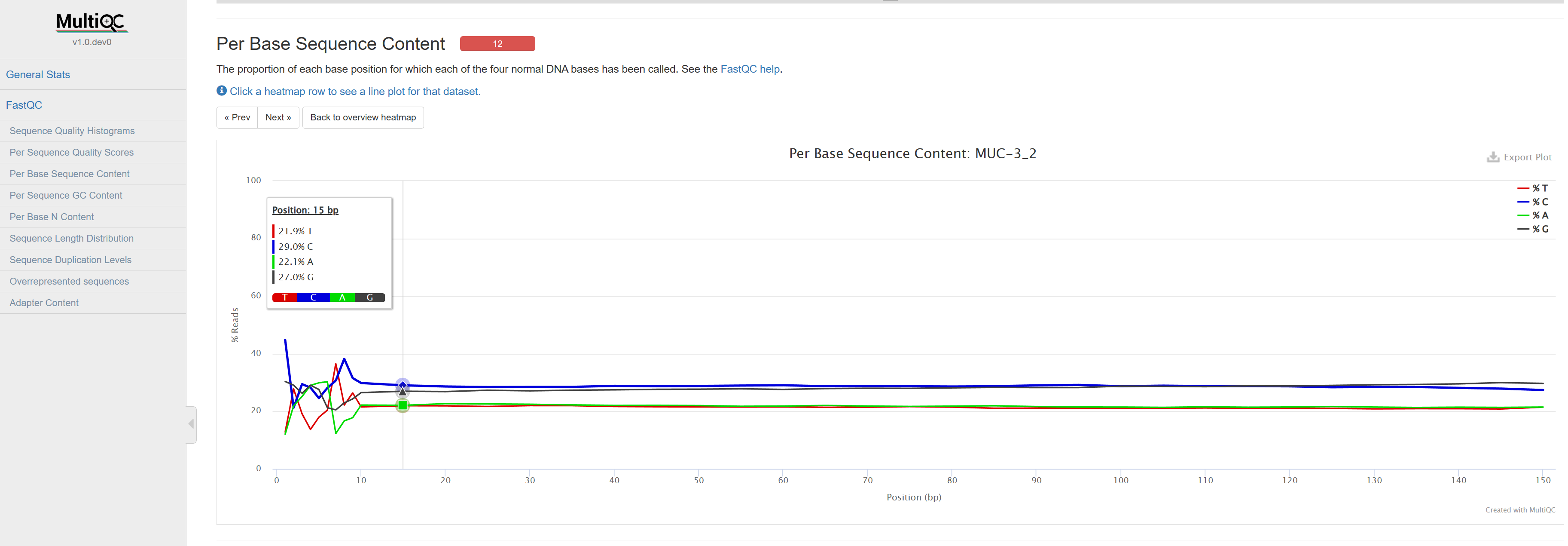

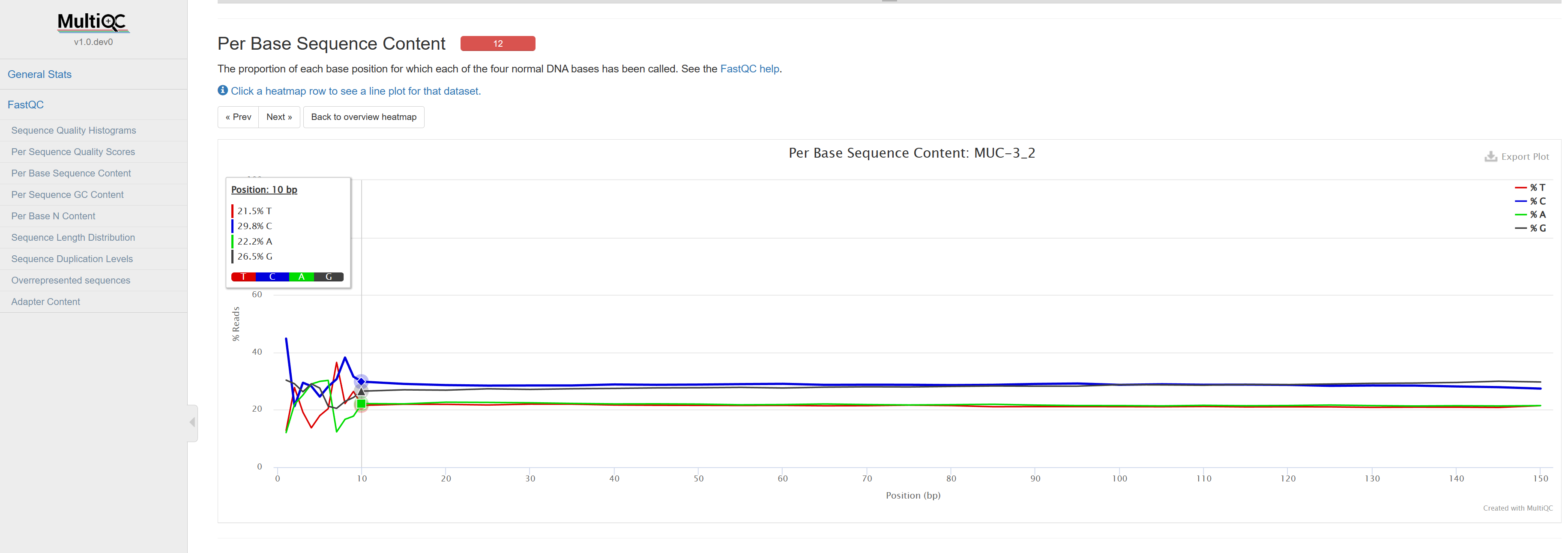


A

B

**Supplementary Fig. 21** The information of ATCG base proportion curves in the location of FOC and TES.

**Supplementary Table 1** The citation times of each tools in Google Scholar(due to May 4, 2024)

| Stage | Software | Year | Citations |
| --- | --- | --- | --- |
| Quality Control | fastp | 2018 | 12,035 |
|  | Trimmomatic | 2014 | 49,711 |
|  | Cutadapt | 2011 | 25,527 |
|  | Trim_Galore | 2012 | 300 |
| Alignment | Bowtie | 2009 | 23,297 |
|  | Bowtie2 | 2012 | 46,223 |
|  | TopHat | 2009 | 13,278 |
|  | TopHat2 | 2013 | 13,163 |
|  | HISAT | 2015 | 16,603 |
|  | HISAT2 | 2019 | 5,329 |
|  | STAR | 2013 | 28,003 |
| Quantifiction | RSEM | 2011 | 17,533 |
|  | featureCounts | 2014 | 17,976 |
|  | HTseq | 2015 | 18,865 |
|  | Kallisto | 2016 | 7,869 |
|  | Salmon | 2017 | 7,944 |
| DE Analysis | DESeq2 | 2014 | 63,980 |
|  | edgeR | 2010 | 35,899 |
|  | limma | 2015 | 28,199 |
|  | bayseq | 2010 | 977 |
|  | NOISeq | 2011 | 1,636 |
|  | Cuffdiff2 | 2013 | 3,801 |
|  | SAMSeq | 2013 | 562 |
| AS Analysis | rMATS | 2014 | 1,936 |
|  | MISO | 2010 | 1,503 |
|  | VAST TOOLS | 2014 | 605 |
|  | DEXeq | 2012 | 1,079 |
|  | Whippet | 2018 | 148 |

**Supplementary Table 2** Summary of the trimming parameters of each data sets

| Data sets | FOC | TES |
| --- | --- | --- |
| M. oryzae | 10bp | 15bp |
| C. gloeosporioides | 10bp | 15bp |
| V. dahliae | 10bp | 12bp |
| P. tomentosa | 10bp | 15bp |
| M. musculus | 9bp | 10bp |
| U. maydis | 10bp | 15bp |
| R. stolonifer | 10bp | 15bp |

**Supplementary Table 3** The Q20 and Q30 base content percentage of filter results obtained by using fastp software under three trimming parameters (raw, FOC, TES).

| Q20 | raw read1 before filtering | foc read1 before filtering | tes read1 before filtering |
| --- | --- | --- | --- |
| Control1 | 99.85% | 100% | 100% |
| control2 | 99.83% | 100% | 100% |
| control3 | 99.82% | 100% | 100% |
| treat1 | 99.91% | 100% | 100% |
| treat2 | 99.91% | 100% | 100% |
| treat3 | 99.90% | 100% | 100% |
|  |  |  |  |
| Q30 | raw read1 before filtering | foc read1 before filtering | tes read1 before filtering |
| control1 | 99.85% | 100% | 100% |
| control2 | 99.83% | 100% | 100% |
| control3 | 99.82% | 100% | 100% |
| treat1 | 99.91% | 100% | 100% |
| treat2 | 99.91% | 100% | 100% |
| treat3 | 99.90% | 100% | 100% |

**Supplementary Table 4** Summary of the AS results using datasets from plant and animal species

| Software | AS Type | Species | |
| --- | --- | --- | --- |
|  |  | P. tomentosa | M. musculus |
| Bowtie2 | SE | 1341 | 18995 |
|  | MXE | 36 | 737 |
|  | A3SS | 4267 | 8347 |
|  | A5SS | 2625 | 5317 |
|  | RI | 2933 | 4104 |
| HISAT2 | SE | 21350 | 80989 |
|  | MXE | 2110 | 13063 |
|  | A3SS | 63936 | 81423 |
|  | A5SS | 40815 | 45234 |
|  | RI | 25883 | 16851 |
| STAR | SE | 48461 | 86730 |
|  | MXE | 8973 | 15915 |
|  | A3SS | 115439 | 83434 |
|  | A5SS | 73924 | 45766 |
|  | RI | 39724 | 16857 |

**Supplementary Table 5** Summary of the DE methods used in this study

|  | normalization | fitting | hypothesis testing |
| --- | --- | --- | --- |
| DESeq2 | ratio | local | Wald |
|  | poscounts | parametric |  |
|  | iterate | mean | LRT |
|  |  | glmGamPoi |  |
| edgeR | TMM | default | ExactTest |
|  | TMMwsp |  |  |
|  | RLE | GLM | glmLRT |
|  | UQ |  |  |
| limma-voom | TMM | voom | eBayes |
|  | TMMwsp |  |  |
|  | RLE |  |  |
|  | UQ |  |  |

**Supplementary Table 6** The results of Kruskal-Wallis rank sum test

| U. maydis | | |  | R. stolonifer | | |
| --- | --- | --- | --- | --- | --- | --- |
|  | chi_squared | p_value |  |  | chi_squared | p_value |
| ratio | 1.842972764 | 0.870418008 |  | poscounts | 2.040027308 | 0.84357924 |
| poscounts | 1.845002096 | 0.870149249 |  | ratio | 2.141498816 | 0.829239819 |
| RLE | 322.715047 | 1.30E-67 |  | none | 159.1802483 | 1.48E-32 |
| UQ | 336.5796977 | 1.36E-70 |  | RLE | 167.779354 | 2.17E-34 |
| TMMwsp | 339.4074513 | 3.34E-71 |  | TMM | 174.9234558 | 6.49E-36 |
| TMM | 342.5261645 | 7.11E-72 |  | TMMwsp | 176.6625347 | 2.76E-36 |
| none | 439.5646836 | 8.75E-93 |  | UQ | 195.5992825 | 2.48E-40 |

**Supplementary Table 7** Summary of the datasets used in this study

| Data sets | BioProject | Reference | Sample | Sample type | Platform | Read Type | Read length | #M seqs | #bps |
| --- | --- | --- | --- | --- | --- | --- | --- | --- | --- |
| M. oryzae | PRJNA523930 | GCA_000002495.2 | MUC | MoCMP1 | Illumina HiSeq 4000 | PE | 150bp | 31.47M | 9.44G |
|  |  |  | MUT | MoCMP1 under Mn treat | Illumina HiSeq 4000 | PE | 150bp | 29.6M | 8.88G |
| C. gloeosporioides | PRJNA391239 | GCA_000319635.1 | STJ16 | W16 (STJ16 replicate) | Illumina HiSeq 4000 | PE | 150bp | 35.48M | 8G |
|  |  |  | FZ | W16(FZ replicate) | Illumina HiSeq 4000 | PE | 150bp | 33.9M | 6.76G |
| V. dahliae | PRJNA1109783 | GCA_000150675.2 | CK | XS11 | BGISEQ-500 | PE | 150bp | 31.5M | 3G |
|  |  |  | ZT | XS11 under Lipopeptide mixture | BGISEQ-500 | PE | 150bp | 30.8M | 3.1G |
|  |  |  | XS | XS11 | Illumina HiSeq 2500 | PE | 150bp | 23.2M | 3.9G |
| P. tomentosa | PRJNA561520 | GCA_000002775.4 | L1 | mature leaf day 1 | Illumina HiSeq 2000 | PE | 150bp | 25.8M | 7.74G |
|  |  |  | L10 | mature leaf day 10 | Illumina HiSeq 2000 | PE | 150bp | 25.8M | 7.65G |
| M. musculus | PRJNA886709 | GRCm38 | SHAM | sham-operated | Illumina NovaSeq 6000 | PE | 150bp | 25.9M | 7.81G |
|  |  |  | TBI | traumatic brain injury | Illumina NovaSeq 6000 | PE | 150bp | 23.9M | 7.2G |
| U. maydis | PRJNA998905 | Umaydis521_2.0.58 | Control | Wildtype | Illumina HiSeq 4000 | PE | 150bp | 20.6M | 6.2G |
|  |  |  | Treat | Pcrg1:grx4 | Illumina HiSeq 4000 | PE | 150bp | 21.8M | 6.5G |
| R. stolonifer | PRJNA940265 | GCA_003325415.1 | Control | Control blank | Illumina NovaSeq 6000 | PE | 150bp | 30.9M | 9.3G |
|  |  |  | Treat | Minimum inhibitory concentration | Illumina NovaSeq 6000 | PE | 150bp | 35.3M | 10.7G |

**Supplementary Table 8** Summary of the tools used in this study

| Software | Stage | Version |
| --- | --- | --- |
| Filtering and trimming | fastp | 0.22.0 |
|  | Trim_Galore | 0.6.10 |
| Quality Control | FastQC | 0.12.1 |
| Read mapping | HISAT2 | 2.1.0 |
|  | STAR | 2.7.10b |
|  | Bowtie2 | 2.2.5 |
| Quantification | featureCounts | 2.0.1 |
|  | RSEM | 1.2.28 |
|  | Salmon | 0.14.1 |
| DE analysis | DESeq2 | 1.42.0 |
|  | edgeR | 4.0.5 |
|  | limma-voom | 3.58.1 |
| AS analysis | rMATS | 3.1.0 |
|  | Whippet | 1.6.2 |
|  | SpliceWiz | 1.5.2 |
